# IL-22 produced by type 3 innate lymphoid cells (ILC3s) reduces the mortality of type 2 diabetes mellitus (T2DM) mice infected with Mycobacterium tuberculosis

**DOI:** 10.1371/journal.ppat.1008140

**Published:** 2019-12-06

**Authors:** Deepak Tripathi, Rajesh Kumar Radhakrishnan, Ramya Sivangala Thandi, Padmaja Paidipally, Kamakshi Prudhula Devalraju, Venkata Sanjeev Kumar Neela, Madeline Kay McAllister, Buka Samten, Vijaya Lakshmi Valluri, Ramakrishna Vankayalapati

**Affiliations:** 1 Department of Pulmonary Immunology, Center for Biomedical Research, The University of Texas Health Science Center, Tyler, Texas, TX, United States of America; 2 Immunology and Molecular Biology Department, Bhagwan Mahavir Medical Research Centre, Hyderabad, Telangana, India; Portland VA Medical Center, Oregon Health and Science University, UNITED STATES

## Abstract

Previously, we found that pathological immune responses enhance the mortality rate of *Mycobacterium tuberculosis* (*Mtb*)-infected mice with type 2 diabetes mellitus (T2DM). In the current study, we evaluated the role of the cytokine IL-22 (known to play a protective role in bacterial infections) and type 3 innate lymphoid cells (ILC3s) in regulating inflammation and mortality in *Mtb*-infected T2DM mice. IL-22 levels were significantly lower in *Mtb*-infected T2DM mice than in nondiabetic *Mtb*-infected mice. Similarly, serum IL-22 levels were significantly lower in tuberculosis (TB) patients with T2DM than in TB patients without T2DM. ILC3s were an important source of IL-22 in mice infected with *Mtb*, and recombinant IL-22 treatment or adoptive transfer of ILC3s prolonged the survival of *Mtb*-infected T2DM mice. Recombinant IL-22 treatment reduced serum insulin levels and improved lipid metabolism. Recombinant IL-22 treatment or ILC3 transfer prevented neutrophil accumulation near alveoli, inhibited neutrophil elastase 2 (ELA2) production and prevented epithelial cell damage, identifying a novel mechanism for IL-22 and ILC3-mediated inhibition of inflammation in T2DM mice infected with an intracellular pathogen. Our findings suggest that the IL-22 pathway may be a novel target for therapeutic intervention in T2DM patients with active TB disease.

## Author summary

Type 2 diabetes mellitus (T2DM) during *Mtb*-infection leads to a pathological immune response in the lung. IL-22 plays an important role in the host defense against bacteria at mucosal surfaces, maintaining tissue barrier integrity by protecting epithelial cells and reducing chronic inflammation. In the current study, we investigated the role of IL-22 during *Mtb* infection in human samples and T2DM mice. We found that plasma IL-22 levels were significantly lower in *Mtb*-infected T2DM mice than in *Mtb*-infected nondiabetic mice. Similarly, IL-22 levels were significantly lower in TB patients with T2DM than in TB patients without T2DM. Type 3 innate lymphoid cells (ILC3s) were an important source of IL-22 in the lungs of *Mtb*-infected mice. *Mtb*-infected T2DM mouse lungs had fewer IL-22+ ILC3s than did the lungs of *Mtb*-infected non-T2DM mice. Recombinant IL-22 and IL-22 producing ILC3s reduced the bacterial burden, lung epithelial cell damage, insulin level and lipid metabolites and prolonged the survival of *Mtb*-infected T2DM mice. Our findings demonstrate that IL-22 produced by ILC3s is essential to inhibit excess inflammation and epithelial cell damage in T2DM mice infected with *Mtb*. Further understanding of these mechanisms and human studies can help to treat TB patients with T2DM.

## Introduction

Type 2 diabetes mellitus (T2DM) individuals are susceptible to various bacterial, viral and protozoan infections, including *Mycobacterium tuberculosis* (*Mtb*) [1–3]. Enhanced susceptibility to *Mtb* infection in patients with T2DM has been attributed to several factors, such as direct effects related to hyperglycemia and insulin resistance and indirect effects related to macrophage and lymphocyte function [4–7]. T2DM significantly increases the risk of developing active tuberculosis (TB) disease in latent tuberculosis infection (LTBI)+ individuals [8]. TB patients with T2DM are unresponsive to TB therapy which leads to treatment failure, relapse, and death [9,10]. Abnormal physiological pulmonary functions of T2DM individuals delay *Mtb* clearance [11]. Patients with chronic T2DM show impaired innate and adaptive immune responses to *Mtb* antigens [7,12,13].

Among the various immune cells, macrophages play an important role in defense against *Mtb* infection by producing cytokine and chemokines and promoting granuloma formation to prevent *Mtb* dissemination [14,15]. Monocytes from T2DM individuals express an increased level of CCR2, which influences the migratory capacity of monocytes [16]. TB patients with T2DM exhibit an increased frequency of hypodense alveolar macrophages, which correlate with disease severity [17]. Neutrophilic inflammation is a central feature of TB patients with T2DM and is associated with macrovascular complications [4]. Individuals with T2DM and TB exhibit an increased level of systemic β-defensin, and excess production of antimicrobial peptides such as cathelicidin (LL37) and human beta defensin- 2 which can initiate tissue damage [18]. T2DM also influences adaptive immunity against *Mtb* infection [13], and patients with T2DM-TB exhibit elevated frequencies of IFN-γ, IL-2, TNF-α and IL-17 producing CD4+ cells [7,19]. Previously, we found that natural killer (NK) and CD11c+ cell interactions can increase IL-6 production, which in turn drives the pathological immune response and mortality associated with *Mtb* infection in diabetic mice [19]. However, the various immune cell populations and factors that can inhibit pathological immune responses in TB patients with T2DM are not known.

Innate lymphoid cells (ILCs) play an important role in controlling infections and maintaining homeostasis by regulating excess inflammation [20–22]. These cells mirror the phenotypes and functions of T cells but do not have antigen-specificity [20,23]. ILCs are divided into three groups based on the expression of transcription factors, cytokines production and cytotoxic function [21]. ILC1s express T-bet and produce interferon-γ (IFN-γ). ILC2s express higher levels of GATA-3 and produce IL-5, IL-13 and amphiregulin. ILC3s express the transcription factor *RORγt* and produce the Th17 cytokines IL-17 and IL-22. ILC3s accumulate rapidly in the lung after *Mtb* infection and mice that lack ILC3s are unable to control *Mtb* growth [24]. IL-17 and IL-22 are produced by ILC3s control the early stage of *Mtb* infection [24].

The Th17 cytokine IL-22 inhibits the intracellular growth of *Mtb* [25], provide protection during virulent *Mtb* infection [26,27] and regulates vaccine-induced protective immunity against challenge with *Mtb* [28]. IL-22 contributes to tissue healing processes and promotes innate immune responses to limit damage during infections [29,30]. IL-22 is primarily produced by innate lymphoid cells (ILCs), NK cells, macrophages, NKTs, activated Th1, Th17, Th22 and γδ T cells [26,31]. IL-22 binds to its heterodimeric receptor complex consisting of IL-22R1 and IL-10R2 to activate the JAK-STAT signaling pathways [32,33]. IL-22R is expressed by epithelial cells, dermal cells, liver, pancreas, and kidney cells [33].

Serum IL-22 levels are significantly reduced in TB patients with T2DM compared with TB patients without T2DM [1,34], however limited information is available regarding the role of IL-22 during TB-diabetes comorbidity [35]. In a high-fat diet mouse model of T2DM, the impaired induction of IL-22 by CD4+ cells enhances susceptibility to infection by the intestinal pathogen *Citrobacter rodentium* [36] The intraperitoneal administration of IL‐22 significantly improve host mucosal defense and hyperglycemia and dyslipidemia in T2DM mice [36].

IL‐22 has been shown to protect insulin-producing β-cells by suppressing endoplasmic reticulum stress and inflammation, restoring glucose homeostasis, preserving the integrity of the gut mucosal barrier, and improving endotoxemia and chronic inflammation in an obesity-induced T2DM mouse model [36,37]. It is not known whether IL-22 and ILC3 cells can regulate pathological immune responses during *Mtb* infection in hosts with T2DM. In this study, we determined the contribution of ILC3 cells and IL-22 in reducing T2DM-induced pathology and survival of *Mtb*-infected T2DM mice. We also determined the IL-22-dependent mechanisms that reduce pathology in the lungs of T2DM mice infected with *Mtb*.

## Methods

### Patient population

Blood samples were obtained from 32 patients with active TB and 14 patients with both active TB and T2DM. All included patients were newly diagnosed and had culture proven active pulmonary TB. Patients with any prior episode of TB treatment, pregnancy, seropositive for HIV or taking immunosuppressive drug were excluded from the study. Demographic details of the patients are provided in S1 Table.

### Collection of blood sample and plasma separation

Whole blood samples were collected by venipuncture in a citrate-treated tubes. Plasma samples were immediately separated by refrigerated centrifugation for 15 minutes at 2,000 x g. The cell free plasma from the blood samples was stored at -70°C.

### Animals

Specific-pathogen-free 6-week-old female wild-type C57BL/6 mice were purchased from Jackson Laboratory and housed at the animal facility of the University of Texas Health Science Center at Tyler. All mice were maintained and used in accordance with approved protocol of The Institutional Animal Care and Use Committee of the University of Texas Health Science Center at Tyler. Sample sizes were chosen following empirical statistical power analysis based on previous published studies [19]. For survival studies 10 mice/group were used, while for immune-phenotyping and histological analysis 5 mice/group were used. All mice were maintained on a standard rodent chow diet (LabDiet, catalog number 5053, St. Louis, MO, USA: 4.07 kcal/gm) during the experiment. After *Mtb* infection all mice were housed at five animals per cage in high-efficiency particulate air (HEPA) filtered racks in certified animal biosafety level 3 (ABSL-3) laboratories. The weights and blood glucose levels of mice were recorded weekly.

### Ethics statement

All human studies were approved by the Institutional Review Board of the Bhagwan Mahavir Medical Research Centre, and informed written consent was obtained from all participants. All human subjects involved in our study were adults. All animal studies were approved by the Institutional Animal Care and Use Committee of the University of Texas Health Science Center at Tyler (Protocol #587). All animal procedures involving the care and use of mice were undertaken in accordance with the guidelines of the NIH/OLAW (Office of Laboratory Animal Welfare).

### Induction of type 2 diabetes

Type 2 diabetes was induced by the combined administration of streptozotocin (STZ) and nicotinamide (NA). STZ was dissolved in a 50 mM citric acid buffer, and 180 mg/kg body weight was administered intraperitoneally thrice at 10 day intervals. NA was dissolved in saline, and 60 mg/kg body weight was administered intraperitoneally 15 minutes before the administration of STZ. Blood glucose was measured using a glucometer at weekly intervals for up to 8 months. Mice were considered diabetic if their blood glucose was >250 mg/dl. Blood glucose levels of the control mice remained between 80 and 100 mg/dl [19].

### Aerosol infection of mice with Mtb H37Rv

Before infecting mice with *Mtb* H37Rv, bacteria were grown in 7H9 liquid medium to mid-log phase and then frozen in aliquots at -70°C. Bacterial counts were determined by plating on 7H10 agar plates supplemented with oleic albumin dextrose catalase (OADC). For infection, bacterial stocks were diluted in 10 ml of normal saline to 0.5 x 10^6^ colony-forming units (CFU)/ml, 1 x 10^6^ CFU/ml, 2 x 10^6^ CFU/ml and 4 x 10^6^ CFU/ml and placed in a nebulizer in an aerosol exposure chamber custom made by the University of Wisconsin. In preliminary studies, groups of three mice were exposed to the aerosol at each concentration for 15 minutes. After 24 hours, the mice were euthanized, and homogenized whole lungs were plated on 7H10 agar plates supplemented with OADC. Colony forming units were determined after 22 days of incubation at 37°C with 5% CO_2_. For further studies, we selected the concentration that deposited ~100 bacteria in the lung during aerosol infection.

### Lung cell preparation

Lungs were harvested from the control or T2DM mice at the indicated time points after *Mtb* challenge and were placed into 30-mm dishes containing 2 ml of Hank's balanced salt solution (HBSS). The tissues were minced with scissors into pieces no larger than 2–3 mm, and the fluid was discharged onto a 70-μm filter (BD Biosciences, San Jose, CA) that had been prewetted with 1 ml of PBS containing 0.5% bovine serum albumin (BSA, Sigma-Aldrich) suspended over a 50-ml conical tube. The syringe plunger was then used to gently disrupt the lung tissue before washing the filter with 2 ml of cold PBS/0.5% BSA.

### Recombinant IL-22 treatment

In some experiments, mice were treated with recombinant IL-22. One month after the induction of T2DM, mice were challenged with aerosolized *Mtb*. Five months p.i., one group of mice were intravenously treated with a single dose of recombinant IL-22 (100 ng/kg body weight) and another group of mice was treated with recombinant IL-22 (100 ng/kg body weight) (BioLegend) or PBS twice a week.

### Isolation of lung epithelial cells

Lungs from *Mtb*-infected mice were mechanically homogenized and filtered through a 70-μm cell strainer. Lung epithelial cells were isolated by negative selection with magnetic beads conjugated to an antibody cocktail (Stem Cell Inc.), and negatively selected cells were >96% EpCAM^+^, as measured by flow cytometry.

### Isolation of type 3 innate lymphoid 3 cells (ILC3s) and adoptive transfer

ILC3 populations, lymphoid tissue inducer (LTi) (Lin-CD127+NK1.1-NKp46-CCR6+) and NCR+ (Lin-CD127+NK1.1-NKp46+CCR6-) cells, were sorted from the pooled spleen, lung, intestine and peripheral lymph node cells of CD45.1 C57BL/6 mice by using a FACSAria cytometer (BD Biosciences). The purity of the flow cytometry-sorted cell population was greater than 98%. Flow-sorted LTi or NCR+ cells were adoptively transferred (0.5 X 10^5^) through tail vein injection to CD45.2 *Mtb*-infected T2DM mice.

### In vivo intestinal barrier function assay

Control or *Mtb*-infected T2DM mice were treated with PBS or recombinant IL-22 and gavaged with 10 mg/ml 10 kDa FITC-dextran (40 mg/100 g body weight) (Sigma Aldrich). Twelve hours later, whole blood was collected, and serum was separated by centrifugation at 3,000 rpm for 10 minutes. Serum was then diluted 4-fold in PBS and added to a 96-well black wall microplate in duplicate for measurement of fluorescence intensity using a fluorometer (BioTek) [38].

### Antibodies and other reagents

For flow cytometry, we used PE anti-IL-22 (eBioscience), FITC anti-lineage (Biolegend), PE anti-NKp46 (Biolegend), PE cy7 anti-CCR7 (Biolegend), PE cy7 anti-CD127 (Biolegend), Brilliant violet 510 anti-Thy1.1, PE anti-ROR-γ (BD biosciences), PE anti-GATA-3 (Biolegend), Brilliant violet 640 anti-ICOS (Biolegend), APC anti-CD4 (Biolegend), APC anti-γδ T cells (eBioscience), PerCP5.5 anti-NK1.1 (Biolegend) and PE anti-CD8 (Biolegend). Streptozotocin and nicotinamide were obtained from Sigma Chemicals. Anti-EpCAM, anti-Ly6G, anti-F4/80, anti-CD11C and secondary antibodies (goat anti-hamster IgG-Alexa 568, donkey anti-rat-Alexa 488, goat anti-rabbit-Alexa 647) and DAPI were obtained from life technologies for confocal microscopy. γ-Irradiated *Mtb* H37Rv (γ-*Mtb*) was obtained from BEI Resources. Highly purified mouse recombinant IL-22 with a specific activity of 1 × 10^8^ units/mg was purchased from Biolegend, USA (Bedford, MA).

### Measurement of serum insulin concentrations

After 5 months of *Mtb* infection T2DM mice were given PBS or treated with recombinant IL-22. After one month of treatment, serum insulin levels were measured using a Mercodia Ultrasensitive Insulin ELISA Kit (Mercodia AB Uppsala, Sweden).

### Measurement of serum lipid profiles

*Mtb* infected T2DM mice were administered PBS or treated with recombinant IL-22 or ILC3s. After one month, serum free fatty acid (FFA), cholesterol and triglyceride levels were measured using either a fluorometric or a colorimetric assay (Cayman Chemicals, USA) according to the manufacturer’s instructions.

### Flow cytometry and intracellular staining

After the mice were euthanized, their lungs were perfused through the right ventricle with 5 ml of PBS. Lungs were mechanically homogenized and filtered through a 70-μm cell strainer. The red blood cells were lysed using BD Pharm Lyse (BD Biosciences). Surface staining for leukocyte populations was performed. For IL-22 and IFN-γ intracellular staining, cells were suspended at 10^6^ cells/ml in RPMI 1640 containing 10% FBS and brefeldin A (5 μg/ml) in 24-well culture plates and stimulated with PMA (1 μg/ml) and ionomycin (1 μg/ml), after which all cells were incubated for another 4 hours at 37°C to allow for the intracellular accumulation of cytokines. Cells were permeabilized with 0.1% saponin and stained for intracellular IL-22 and IFN-γ. The cells were washed and resuspended in FACS buffer. Cells were analyzed by flow cytometry using a FACS Calibur flow cytometer.

### Gating strategies

Type 1 innate lymphoid cells (ILCs1) were identified as CD45+CD127+lin-NKp46+NK1.1+ cells. Type 2 innate lymphoid cells (ILCs2) were gated as CD45+CD127+lin-Rorγt-Sca1+ cells. In Type 3 innate lymphoid cells, tissue lymphoid inducer (LTi) cells were gated as CD45+CD127+lin-NK1.1-Rorγt+NKp46-CCR6+ cells and NCR+ cells were gated as CD45+CD127+lin-NK1.1-Rorγt+NKp46+CCR6- cells.

### Real-time PCR for the quantification of cytokine mRNA

Total RNA was extracted from lung epithelial cells as described previously [39]. Total RNA was reverse transcribed using the Cloned AMV First-Strand cDNA Synthesis Kit (Life Technologies). Real-time PCR was performed using the QuantiTect SYBR Green PCR Kit (Qiagen) in a sealed 96-well microtiter plate (Applied Biosystems) on a spectrofluorometric thermal cycler (7700 PRISM; Applied Biosystems). PCRs were performed in triplicate as follows: 95°C for 10 min and 45 cycles of 95°C for 15 s, 60°C for 30 s, and 72°C for 30 s. All samples were normalized to the amount of β-actin/GAPDH transcript present in each sample. The primers used in the study are provided in S2 Table.

### Histology and immunohistochemistry

At each time point, mice were euthanized, and the harvested lungs were placed in 10% neutral buffered formalin (Statlab, McKinney, TX, USA) for 48 hours to inactivate the infectious agent. Paraffin-embedded blocks were cut into 5 μm-thick sections. For morphometric lesion analyses, the lung sections were stained with hematoxylin and eosin (H&E) and examined in a blinded manner to assess the necrotic lesions. A semi quantitative analysis was performed using a score from 0 (no inflammation) to 4 (severe inflammation) for each of the following criteria: alveolar wall inflammation; alveoli destruction; leukocyte infiltration and perivascular inflammation. Two investigators, independently assessed the immunohistochemical readouts using morphometric analyses.

### Confocal microscopy

Confocal microscopy was performed to colocalize IL-22R1 expressing EpCAM positive cells, Ly6G+, F4/80 and CD11c+ cells in the above lung sections. Nonspecific binding was blocked with 1% goat serum in PBS for 30 minutes. The slides were incubated at 4°C overnight with monoclonal hamster monoclonal anti-CD11c (Abcam), rabbit polyclonal anti-EpCAM (Abcam) and rat monoclonal anti-IL-22R1 (R&D) antibodies. Subsequently, the slides were washed thoroughly using 1X PBS. Then, cells were stained with their respective secondary antibodies (goat anti-hamster IgG-Alexa 568, goat anti-rabbit-Alexa 647 and donkey anti-rat- Alexa 488, Life Technologies). The cells were washed with PBS and mounted with Prolong Gold antifade reagent with DAPI (Life Technologies, USA). The slides were examined and analyzed using a laser-scanning confocal microscope system (Zeiss LSM 510 Meta laser-scanning confocal microscope).

### Statistical analysis

Data analyses were performed using GraphPad Prism (GraphPad Software, Inc., La Jolla, CA). The results are shown as the mean ±SD. For data that were normally distributed, comparisons between groups were performed using a paired or unpaired t-test and ANOVA, as appropriate. Statistically significant differences between two clinical groups were analyzed using the nonparametric Mann–Whitney U-test.

## Results

### T2DM inhibits IL-22 production during Mtb infection

**Fig 1:**
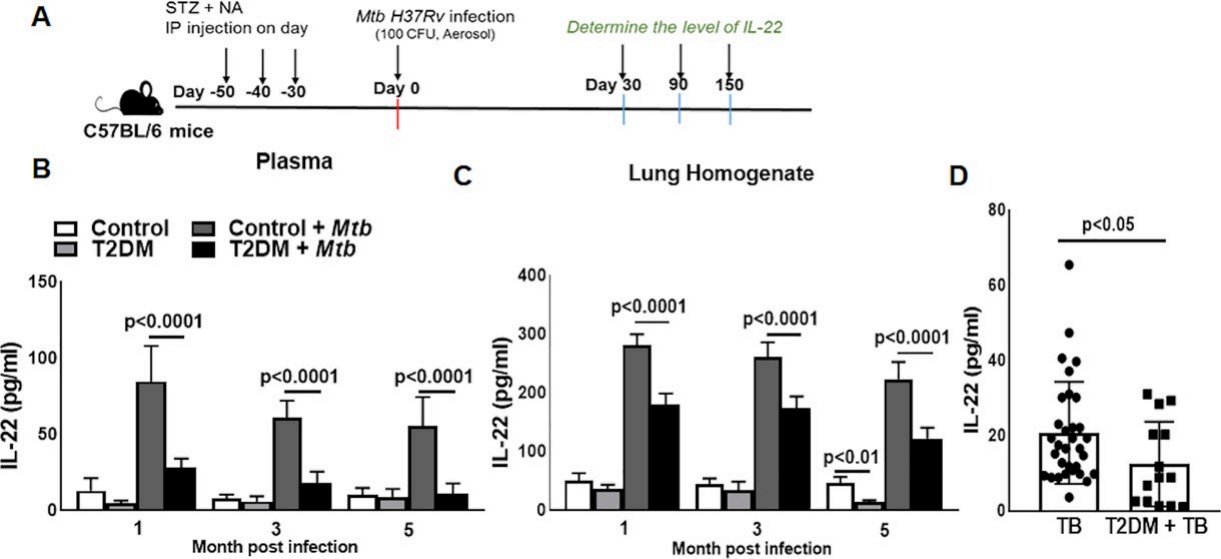
T2DM inhibits IL-22 production during *Mtb* infection. (A) T2DM induced in C57BL/6 mice with the intraperitoneal injection of streptozotocin (180 mg/kg body weight) and nicotinamide (60 mg/kg body weight) as described in the methods section. Control C57BL/6 or T2DM mice were infected with ~100 CFU of aerosolized *Mtb*. A schematic representation of T2DM induction and *Mtb* infection is shown. One, three, and five months after *Mtb* infection, the level of IL-22 was measured in the (B) plasma and (C) lung homogenate by ELISA. (D) Blood plasma samples were obtained from 32 patients with active pulmonary tuberculosis (TB) and 14 patients with TB and type 2 diabetes mellitus (T2DM). The level of IL-22 was determined by ELISA. Five mice per group were used. The mean values, SDs and p-values are shown.

We used a previously published experimentally induced T2DM model for the current investigation (described in the methods section) [19]. One month after the induction of T2DM, control and T2DM mice were challenged with *Mtb* as shown in the schematic diagram (Fig 1A) and described in the methods section. One, three and five months post-*Mtb* infection, IL-22 levels in plasma and lung homogenate were significantly lower in *Mtb*-infected T2DM mice than in *Mtb*-infected nondiabetic control mice (Fig 1B and 1C). We also compared plasma IL-22 levels of TB patients with or without T2DM. As shown in Fig 1D, plasma IL-22 levels were significantly lower in patients with active TB and T2DM than in patients with TB without T2DM (20.80 ± 13.55 *vs*. 12.50 ± 11.24, p<0.05, Fig 1D).

### Type 3 innate lymphoid cells produce IL-22 during Mtb infection

**Fig 2:**
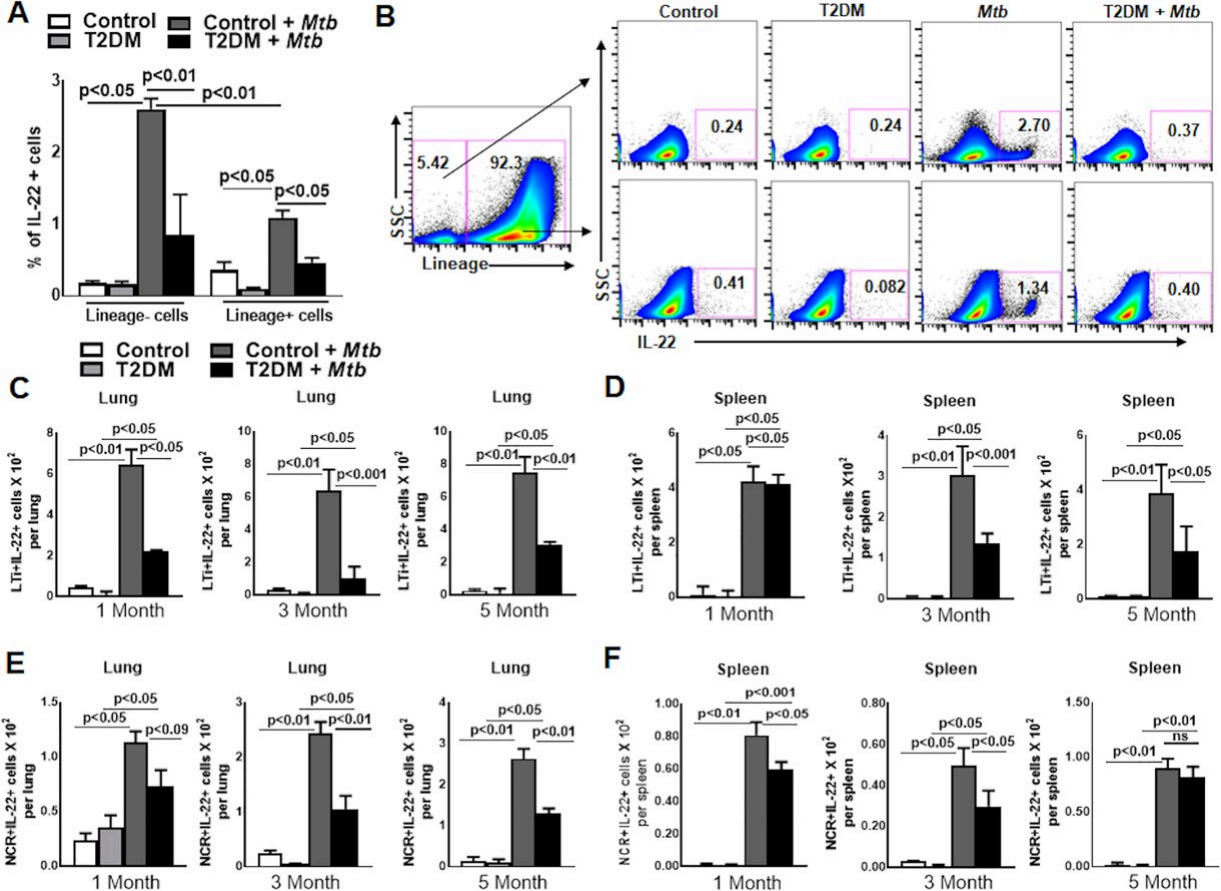
Type 3 innate lymphoid 3 cells (ILC3s) produce IL-22 during *Mtb* infection. Control C57BL/6 and T2DM mice were infected with *Mtb* as shown in Fig 1. (A) One month after *Mtb* infection, the percentage of lineage+IL-22+ and lineage-IL-22+ cells was determined by flow cytometry. (B) A representative flow cytometry plot is shown. (C-F) One, three and five months after *Mtb* infection, the absolute number of (C) lung LTi+IL-22+ cells, (D) splenic LTi+IL-22+ cells, (E) lung NCR+IL-22+ cells, and (F) splenic NCR+IL-22+ cells were determined by flow cytometry. Five mice per group were used. The mean values, SDs and p-values are shown.

To determine the cellular source of IL-22, control and T2DM mice were challenged with aerosolized *Mtb* H37Rv as described in the methods section. We found that *Mtb* infection significantly increased the accumulation of lineage- and lineage+ IL-22 producing cells in the lung of control (lineage- cells: 0.18 ± 0.02 *vs*. 2.61 ± 0.08, p<0.01; lineage+ cells 0.36 ± 0.06 *vs*. 1.09 ± 0.05, p<0.01, Fig 2A) and T2DM mice (lineage- cells: 0.16 ± 0.02 *vs*. 0.85 ± 0.32, p<0.01; lineage+ cells: 0.09 ± 0.01 *vs*. 0.46 ± 0.03, p<0.01, Fig 2A). One month after *Mtb* infection, the lungs of control mice had 3.0-fold higher percentage of lineage-IL-22+ (2.61 ± 0.08 *vs*. 0.85 ± 0.32, p<0.01) and 2-fold higher lineage+IL-22+ (1.09 ± 0.05 *vs*. 0.46 ± 0.03, p<0.01) cells compared with the lungs of T2DM mice (Fig 2A and 2B). We further characterized the lineage+ and lineage- IL-22 producing cells in the major lymphoid organs of the control and T2DM mice after one, three and five months of *Mtb* infection. As shown in S1 Fig, among lineage+ lung cells, CD4+, CD8+, NK cells and γδ+ T cells produced IL-22, but the differences were not significant between *Mtb*-infected control and T2DM mice at one, three and five months post *Mtb* infection. Among lineage- cells, we were unable to detect any IL-22-producing type 1 innate lymphoid cells (ILC1) or type 2 innate lymphoid cells (ILC2) in various lymphoid organs of *Mtb*-infected control and T2DM mice at all three time points (one, three and five months after *Mtb* infection) (S2, S3A and S3B Figs) However, a significant number of lung and splenic ILC1s from *Mtb*-infected T2DM mice were positive for IFN-γ compared with those from the control *Mtb*-infected mice (S2, S3A and S4 Figs). In addition the number of ILC2s were reduced in the lung of *Mtb*-infected T2DM mice compared with control *Mtb*-infected mice at one, three and five months after infection (p<0.05, S3 and S5 Figs). Among the lineage -ve cell population, two subpopulations of ILC3s, both lymphoid tissue inducer (LTi) CD45+CD127+lin-Rorγ+NKp46-CCR6+ cells (50%) and NCR+ (CD45+CD127+lin- Rorγ+NKp46+CCR6-) cells (18%), were positive for IL-22, but the absolute number of the IL-22-producing LTi cells was significantly higher (8-10-fold in lung and 4-fold in spleen, Figs 2C, 2D, S6 and S7) in *Mtb*-infected control mice. IL-22-producing subsets of ILC3s, LTi and NCR+ also accumulate in the lungs of T2DM mice after *Mtb* infection compared to uninfected T2DM mice (p<0.05, Fig 2C–2F). However, as shown in Fig 2C–2F, both subpopulations of IL-22-producing ILC3s were significantly reduced in the lungs and spleens of *Mtb*-infected T2DM mice compared with those of the *Mtb*-infected control mice at one, three and five months after infection (lung LTi+IL-22+ cells: 1 month, p<0.05; 3 months, p<0.001; and 5 months, p<0.001; Fig 2C. Spleen LTi+IL-22+ cells: 1 month, not significant; 3 months, p<0.001; and 5 months, p<0.001; Fig 2D. Lung NCR+ cells: 1 month, not significant; 3 months, p<0.01; and 5 months, p<0.01; Fig 2E. Spleen NCR+ cells 1 month, p<0.05; 3 months, p<0.05; and 5 month, not significant; Fig 2F).

### Recombinant-IL-22 treatment prolongs the survival of Mtb-infected T2DM mice

**Fig 3:**
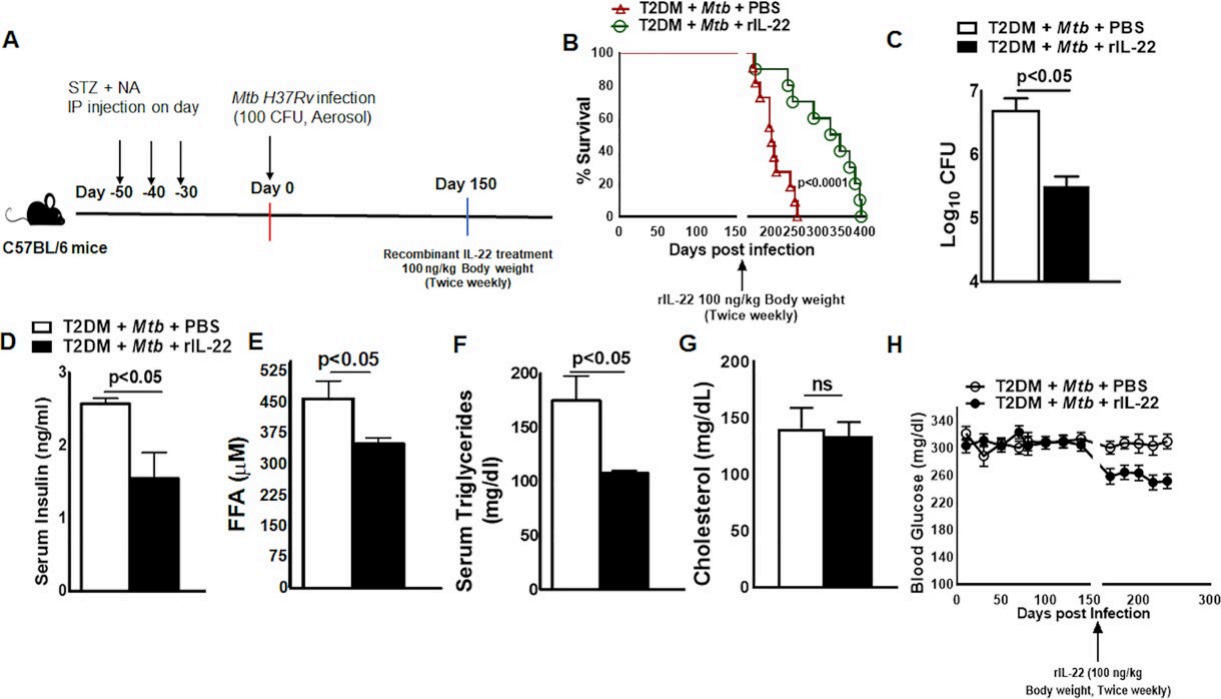
Recombinant-IL-22 treatment prolongs the survival of *Mtb*-infected T2DM mice. One month after the induction of diabetes, T2DM mice were infected with ~100 CFU of aerosolized *Mtb*. Five months after *Mtb* infection, the mice were treated intravenously with recombinant IL-22 (100 ng/kg body weight, twice weekly) or PBS. (A) A schematic representation of *Mtb* infection and recombinant IL-22 treatment in T2DM mice is shown. (B) Survival of *Mtb*-infected T2DM mice treated with recombinant IL-22 or PBS. Survival curves were compared using the log rank test. Data were pooled from two independent experiments (n = 5 mice per group per experiment). (C) Bacterial burden in lungs at 1 month post recombinant IL-22 or PBS treatment. (D) Serum insulin levels were measured by ELISA at 1 month after recombinant IL-22 treatment. (E to G) free fatty acid, triglyceride and Serum cholesterol, levels were measured by biochemical-based assays at 1 month after recombinant IL-22 treatment. (H) Random blood glucose sampling at twenty-day intervals for up to 8 months. Five mice per group were used. The mean values, SDs and p-values are shown.

Based on the above findings, we determined whether treatment with recombinant IL-22 affects survival, bacterial burden and certain insulin-dependent metabolic changes in *Mtb*-infected T2DM mice. Figs 3A and S8 show a schematic representation of *Mtb* infection and recombinant IL-22 treatment in T2DM mice. One month after T2DM induction, the mice were aerosol infected with ~100 CFU of *Mtb*, and at five months p.i., the mice were intravenously treated with a single dose or a twice weekly regimen (up to 35 weeks), of recombinant IL-22 (100 ng/kg body weight) or PBS as described in the methods section. As shown in Figs 3B and S8, 80% of *Mtb*-infected T2DM mice that received PBS died within 6.5 months of *Mtb* infection (median survival, 206.5 days p.i.). In contrast *Mtb*-infected T2DM mice that received a single dose of recombinant IL-22 survived up to 8.2 months (median survival, 206.5 *vs*. 247.5 days, p<0.05). Mice that received recombinant IL-22 (100 ng/kg body weight) twice a week survived up to 11.2 months after *Mtb* infection (median survival, 210.0 *vs*. 336.5 days p.i., p<0.001). Twice a weekly recombinant IL-22 treatment also reduced the bacterial burden in the lungs (Fig 3C). One month after twice a week recombinant IL-22 treatment in *Mtb*-infected T2DM mice, compared with PBS-treated *Mtb*-infected T2DM mice, serum insulin levels were significantly decreased (2.57 ± 0.17 *vs*. 1.55 ± 0.78, p<0.05, Fig 3D), and serum free fatty acids (460.9 ± 93.96 *vs*. 351.1 ± 34.87, p<0.05, Fig 3E) and triglycerides (174.7 ± 51.04 *vs*. 107.6 ± 4.98, p<0.05, Fig 3F) were marginally reduced. However, there was no significant change in blood glucose levels in single or multiple doses of recombinant IL-22 received mice (Figs 3H and S8C).

**Fig 4:**
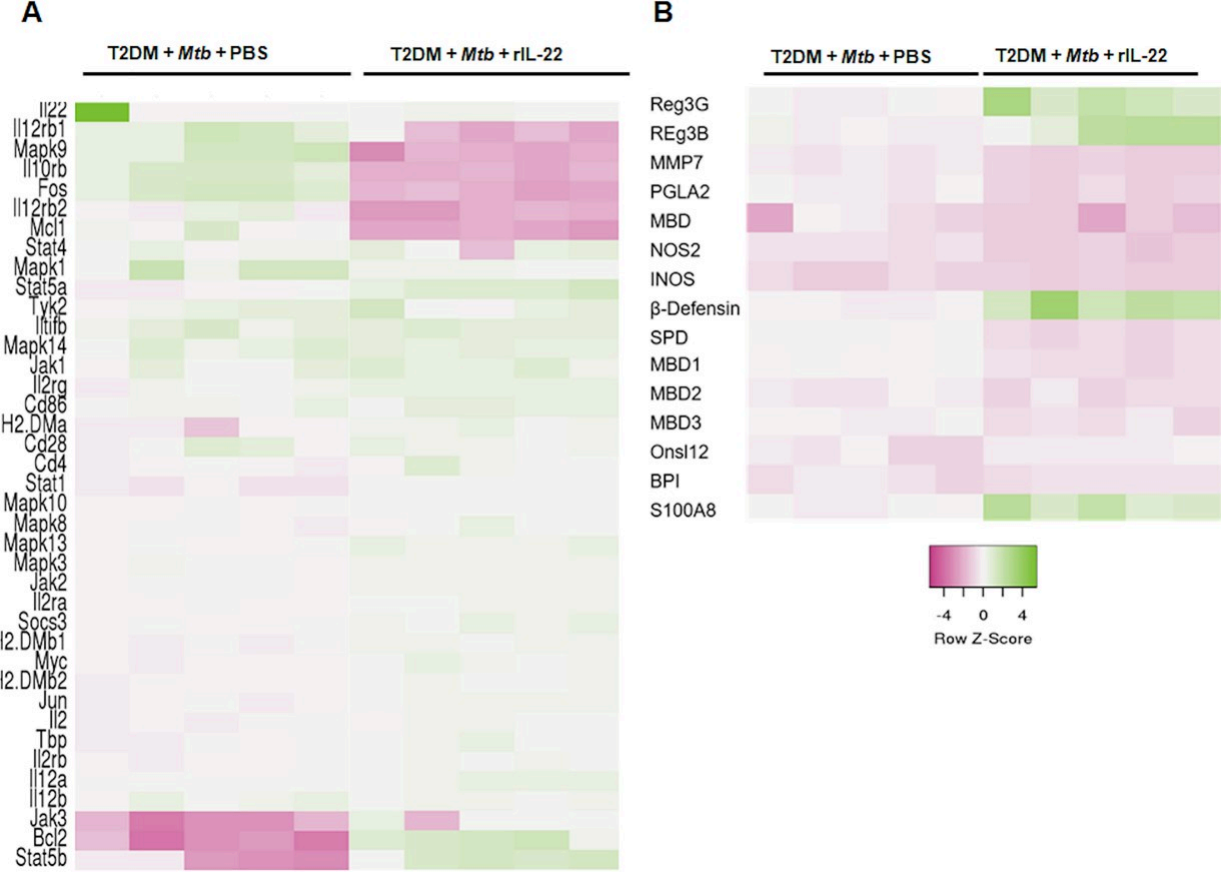
IL-22-mediated signaling enhances the gene expression of anti-apoptotic and antimicrobial proteins in lung epithelial cells. One month after the induction of diabetes, T2DM mice were infected with 50–100 CFU of aerosolized *Mtb*. The mice were then treated intravenously with either recombinant IL-22 (100 ng/kg body weight, twice weekly, starting 5 months p.i.) or PBS. One month after recombinant IL-22 or PBS treatment, lung epithelial cells were isolated by immunomagnetic labeling, and mRNA expression of (A) IL-22 signaling pathway genes and (B) antimicrobial genes were determined by real-time PCR. Five mice per group were used. The mean values, SDs and p-values are shown.

Because IL-22R is highly expressed by epithelial cells [33], in the above groups of mice, lung epithelial cells were isolated, and the expression of genes involved in the IL-22 signaling pathway was determined by PCR. As shown in Fig 4A, the expression of *Bcl2* and *stat5b* was increased, and the expression of *mapk9*, *il-10rb*, *fos*, *il-12rb2*, *mcl1*, *and Stat 4* was decreased in the epithelial cells from the lungs of recombinant IL-22-treated mice (100 ng/kg body weight, twice weekly) (Fig 4A). Recombinant IL-22 treatment also significantly enhanced the expression of genes involved in the production of antimicrobial peptides (Fig 4B).

### IL-22-producing ILC3s enhance the survival of Mtb-infected T2DM mice

**Fig 5:**
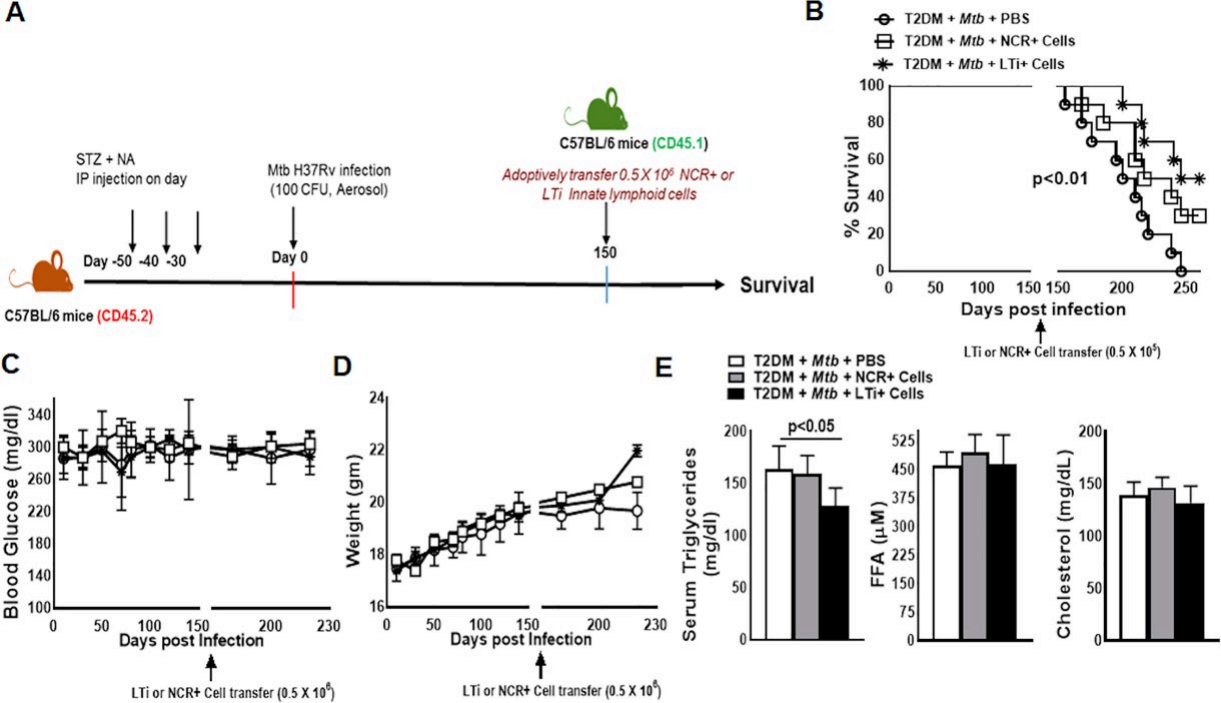
Adoptive transfer of type 3 innate lymphoid cells (ILC3s) enhances the survival of the *Mtb*-infected T2DM mice. T2DM (CD45.2, C57BL/6 background) mice were infected with 50–100 CFU of aerosolized *Mtb*. Five months after *Mtb* infection, 0.5 x 10^5^ NCR+ (Lin-CD127+NK1.1-NKp46+CCR6-) or LTi+ (Lin-CD127+NK1.1-NKp46-CCR6+) pooled cells (from spleen, lung, liver, lymph nodes and mucosal sites) from CD45.1 mice (C57BL/6) were adoptively transferred via tail vein injection (recipient CD45.2 *Mtb*-infected T2DM mice). (A) A schematic representation of T2DM induction, *Mtb* infection and adoptive transfer is shown. (B) Survival of *Mtb*-infected T2DM mice treated with NCR+ or LTi or PBS. Data were pooled from two independent experiments (n = 5 mice per group per experiment). Survival curves were compared using the log rank test (p< 0.001). (C) Blood glucose and (D) weight were measured every 20 days up to 230 days post-*Mtb* infection. (E) Serum free fatty acid, triglyceride and cholesterol, levels at 1 month after adoptive transfer of the ILC3s. Data were pooled from two independent experiments (n = 3 mice per group per experiment). The mean values, SDs and p-values are shown.

As shown in Fig 2, lung ILC3s from *Mtb*-infected T2DM mice produced significantly less IL-22 than those from *Mtb*-infected nondiabetic mice. In the above experiment, we found that recombinant IL-22 treatment enhanced the survival of *Mtb*-infected T2DM mice. In another set of experiments we questioned whether the adoptive transfer of ILC3 subpopulations from control C57BL/6 mice to *Mtb*-infected T2DM mice could prolong their survival. We isolated NCR+ (Lin-CD127+NK1.1-NKp46+CCR6-) and LTi+ cells (Lin-CD127+NK1.1-NKp46-CCR6+) from pooled cells (from spleen, lung, liver, lymph nodes and mucosal sites) of CD45.1 mice (C57BL/6) as mentioned in the methods and shown in Fig 5A. Isolated LTi or NCR+ cells (0.5 x10^5^) were adoptively transferred through tail vein injections into C57BL/6 CD45.2 *Mtb*-infected T2DM mice on the 150^th^ day after *Mtb* infection. As shown in S9 A, adoptively transferred cells were detected in the lung at 15 and 30 days after adoptive transfer. *Mtb*-infected T2DM mice survived up to 205 days, and adoptive transfer of LTi+ cells enhanced their survival up to 253 days (Fig 5B, p<0.01). The adoptive transfer of NCR+ cells also enhanced the survival of *Mtb*-infected T2DM mice (median survival, 205 *vs*. 227 days, p<0.01, Fig 5B). One month after the adoptive transfer of ILC3 subpopulations, there were no significant changes in blood glucose or body weight of *Mtb*-infected T2DM mice (Fig 5C and 5D). In contrast, the level of serum triglycerides was significantly reduced (Fig 5E), and the level of IL-22 increased in the lung and plasma of *Mtb*-infected T2DM mice that received LTi+ cells compared with *Mtb*-infected T2DM mice that received PBS (S10A and S10B Fig).

### IL-22 reduces the severity of lung inflammation and neutrophil-mediated damage of lung epithelial cells in Mtb-infected T2DM mice

**Fig 6:**
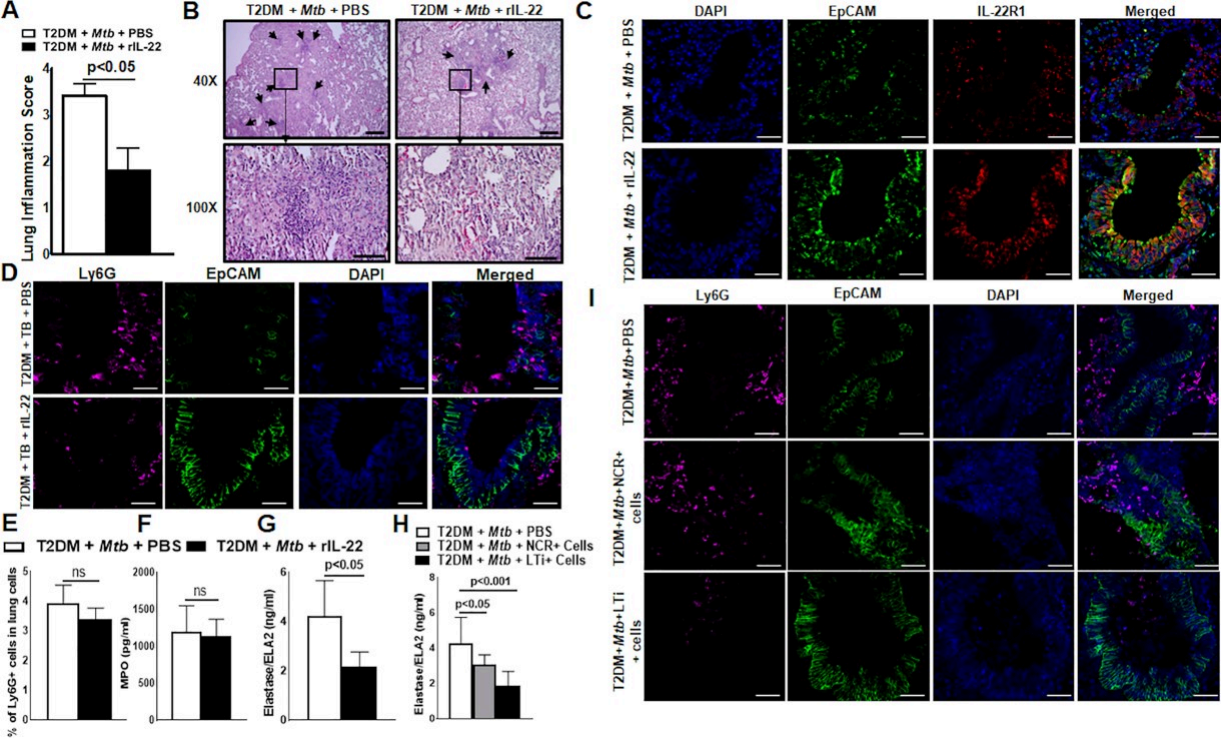
IL-22 reduces the severity of lung inflammation and neutrophil-mediated damage of lung epithelial cells in *Mtb*-infected T2DM mice. One month after the induction of diabetes, T2DM mice were infected with ~100 CFU of aerosolized *Mtb* as shown in Fig 1 and described in the methods section. Five months after *Mtb* infection, T2DM mice were treated intravenously with either recombinant IL-22 (100 ng/kg body weight, twice weekly) or PBS. (A) After one month of recombinant IL-22 treatment, the lungs were isolated and formalin fixed. Paraffin-embedded tissue sections were prepared, and hematoxylin and eosin staining was performed. Lung inflammation scores were quantified by morphometric analysis. (B) Representative hematoxylin and eosin-stained lung tissue sections in multiple fields (at 40X and 100X) are shown. (C) Paraffin-embedded tissue sections were analyzed by confocal microscopy to determine the colocalization of EpCAM-positive cells (green) and IL-22R1+ cells (red). (D) Paraffin-embedded tissue sections were analyzed by confocal microscopy to determine the accumulation of the Ly6G+ cells (magenta) near the EpCAM+ epithelial cell lining (green). (E) The frequencies of Ly6G+ lung cell populations were determined by flow cytometry staining at 1 month after recombinant IL-22 treatment. (F-G) One month after PBS or recombinant IL-22 treatment, lung homogenates of the *Mtb-*infected T2DM mice were collected, and the levels of (F) myeloperoxidase (MPO) and (G) elastase were measured by ELISA. (H and I) T2DM (CD45.2, C57BL/6 background) mice were infected with 50–100 CFU of aerosolized *Mtb*. Five months after *Mtb* infection, 0.5 x 10^5^ NCR+ or LTi+ pooled cells (from spleen, lung, liver, lymph nodes and mucosal sites) from CD45.1 mice (C57BL/6) were adoptively transferred via tail vein injection (recipient CD45.2 *Mtb*-infected T2DM mice). (H) Elastase levels in the lung homogenate were measured by ELISA. (I) Ly6G+ cell accumulation near EpCAM+ cells (epithelial cell lining) in the lungs of ILC3- or PBS-treated *Mtb*-infected T2DM mice was analyzed by confocal microscopy. A representative immunofluorescence image is shown. Five mice per group were used. The mean values, SDs and p-values are shown.

We previously found that *Mtb*-infected T2DM mice had significantly more inflammation than *Mtb*-infected control mice or uninfected T2DM mice [19]. We determined the effect of recombinant IL-22 treatment on the lung pathology of *Mtb*-infected T2DM mice. In the above groups of mice, histological examination of the lungs demonstrated that compared with PBS treatment, recombinant IL-22 treatment significantly reduced lung inflammation (3.40 ± 0.6 *vs*. 1.80 ± 1.10, Fig 6A and 6B). IL-22R1 is mainly expressed by nonhematopoietic cells such as epithelial cells [33,40]. As shown in Fig 6C, in *Mtb*-infected T2DM mice (6 months after infection), the lung epithelial cell lining was severely damaged (disappearance of EpCAM+ cells), and IL-22R1 expression was reduced compared with that of *Mtb*-infected non-T2DM mice. Recombinant IL-22 treatment for 30 days significantly reduced epithelial cell damage and restored IL-22R1 expression (Fig 6C).

Chronic inflammatory responses and ongoing proinflammatory signaling result in the influx of activated phagocytic cells towards the epithelial lining and induce its damage [41–43]. In the above groups of mice, we determined the infiltration of the phagocytic cells by confocal microscopy. As shown in Fig 6D, an increased number of Ly6G+ cells were observed in close proximity to the lung epithelial cell lining of the PBS-treated *Mtb*-infected T2DM mice. IL-22-treated *Mtb*-infected T2DM mice had a reduced accumulation of Ly6G+ cells near the lung epithelial cell lining. However, there was no difference in the recruitment of Ly6G+ cells (the total percentage of lung Ly6G+ cells was similar) in PBS- or rIL-22-treated *Mtb*-infected T2DM mice (Fig 6E). We did not find an accumulation of F4/80+ and CD11c+ phagocytic cells near the lung epithelial cell lining of *Mtb*-infected T2DM mice (S11A Fig). Ly6G+ cells contain cytotoxic substances, neutral proteinases and acid hydrolases, and direct interaction with epithelial cells induces the secretion of acid hydrolases and other enzymes that cause epithelial cell lining damage [44,45]. Five months after *Mtb* infection, we found increased infiltration of the Ly6G+ cells in the lung of T2DM mice compared with those of control mice (p<0.5, S12C Fig). As shown in S11B Fig, in the lungs of *Mtb*-infected T2DM mice (6 months after infection), the epithelial cell lining in the proximity of the Ly6G+ cells was disintegrated (losing membrane integrity) compared with that in the lungs of control mice infected with *Mtb*. We found a significant increase in neutrophil elastase 2 levels in the lung homogenate of *Mtb*-infected T2DM mice compared to that of *Mtb*-infected control mice (S12B Fig). Compared with the PBS control treatment, recombinant IL-22 treatment (1 month, twice weekly) reduced the infiltration of Ly6G+ cells in the epithelial cell lining and significantly reduced neutrophil elastase 2 levels in the lung homogenates of *Mtb*-infected T2DM mice (4.22 ±1.45 ng/ml *vs*. 2.18 ± 0.59 ng/ml, p<0.05; Fig 6G).

We further determined the elastase levels and epithelial cell damage in the lungs of *Mtb*-infected T2DM mice that received PBS or ILC3s. The level of neutrophil elastase 2 was significantly reduced in the lung homogenates of LTi-treated *Mtb*-infected T2DM mice compared with the lung homogenates of *Mtb*-infected T2DM mice (4.27 ± 1.46 *vs*. 1.86 ± 0.8, p<0.001, Fig 6H). Adoptive transfer of NCR+ cells also marginally reduced neutrophil elastase 2 level (4.27 ± 1.46 *vs*. 3.08 ± 0.54, p<0.05, Fig 6H). The adoptive transfer of LTi cells significantly reduced lung epithelial cell lining damage in T2DM mice infected with *Mtb* (Fig 6I).

### IL-22 treatment maintains gut epithelial cell integrity in Mtb-infected T2DM mice

It is known that uncontrolled T2DM can damage the gut epithelial cell lining [46–48]. We determined whether recombinant IL-22 treatment can also prevent gut epithelial cell lining destruction and leakage. Control and T2DM mice were infected with aerosol *Mtb* H37Rv as shown in Fig 1A. At five months p.i., control, T2DM-infected and uninfected mice were orally gavaged with FITC dextran, and then, after 6 hours, FITC levels were measured in the blood. As shown in S13A Fig, FITC levels were significantly increased in T2DM mice compared with that of control mice (30.00 ± 12.00 *vs*. 178.0 ± 28.00, p<0.001). However, *Mtb* infection did not affect the serum FITC levels in the blood of control and T2DM mice (control: 30.00 ± 12.00 *vs*. control + *Mtb*: 50.00 ± 14.00, p = ns; T2DM: 178.0 ± 48.00 *Vs*. T2DM + *Mtb*: 210.0 ± 35.00, p = ns; S13A Fig). Twice weekly treatment with recombinant IL-22 reduced serum FITC levels in *Mtb*-infected T2DM mice (185.31 ± 28.8 *vs*. 141.4 ± 34.8, p<0.05; S13A and S13B Fig). Histological analysis also revealed that the gut epithelial lining of T2DM and *Mtb-*infected T2DM mice was disintegrated compared with that of control and *Mtb*-infected mice, and further treatment of *Mtb*-infected T2DM mice with recombinant IL-22 (twice weekly) for 1 month prevented the gut epithelial cell lining damage (S13C Fig).

## Discussion

IL-22 plays an important role in the host defense against bacteria at mucosal surfaces, maintaining tissue barrier integrity by protecting epithelial cells and reducing chronic inflammation [40,49,50]. T2DM in *Mtb*-infected mice leads to a pathological immune response in the lung and enhances mortality [19]. In the current study, we investigated the role of IL-22 during *Mtb* infection in T2DM mice. We found that plasma IL-22 levels were significantly lower in *Mtb*-infected T2DM mice than in *Mtb*-infected nondiabetic mice. Similarly, IL-22 levels were significantly lower in TB patients with T2DM than in TB patients without T2DM. ILC3s were an important source of IL-22 in the lungs of *Mtb*-infected mice. *Mtb*-infected T2DM mouse lungs had fewer IL-22+ILC3+ cells than the lungs of *Mtb*-infected non-T2DM mice. Recombinant IL-22 and IL-22-producing ILC3+ subpopulations reduced the bacterial burden, lung epithelial cell damage, insulin level and lipid metabolites and prolonged the survival of *Mtb*-infected T2DM mice. Recombinant IL-22 treatment reduced the secretion of neutrophil elastase 2 by epithelial cell-associated neutrophils, which is known to damage epithelial cell lining in the lungs of *Mtb*-infected T2DM mice. Our findings demonstrate that IL-22 produced by ILC3s is essential to inhibit excess inflammation and epithelial cell damage in T2DM mice infected with *Mtb*.

Individuals with T2DM have a threefold increased chance of developing TB, and fifteen percent of the TB burden in the world is associated with T2DM [10,51–53]. T2DM is known to worsen the clinical features of TB and induce severe lung damage [2,54]. We recently found that natural killer (NK) and CD11c+ cell interactions in *Mtb*-infected T2DM mice led to increased IL-6 production, which drove the pathological immune response and reduced survival of *Mtb*-infected T2DM mice [19]. In our published studies, we found that except for IL-22, all pro- and anti-inflammatory cytokine levels (Th1, Th2 and Th17) were significantly higher in the lungs of *Mtb*-infected T2DM mice than in the lungs of nondiabetic *Mtb*-infected mice [19]. Similar findings were noted in the plasma of T2DM TB patients compared with that of TB patients without T2DM [26,55]. Our current findings confirm these findings and further demonstrate that ILC3s are an important source of IL-22 in *Mtb*-infected mouse lungs, and defective IL-22 production by ILC3s in T2DM mice infected with *Mtb* leads to epithelial cell damage and enhanced mortality.

In *Mtb*-infected T2DM mice, recombinant IL-22 treatment or adoptive transfer of ILC3s significantly reduced bacterial burden, reduced inflammation, improved lipid metabolism and prolonged survival in mice. We found that LTi cells, a subpopulation of ILC3, are more efficient than NCR+ cells in reducing inflammation and prolonging the survival of T2DM mice infected with *Mtb*. In addition, recombinant IL-22 treatment significantly alleviated insulin resistance in T2DM mice. IL-22 is known to play an important role in maintaining glucose homeostasis and insulin resistance and restores the mucosal host defense by bacterial clearance in various bacterial infections [36,37]. Our current study demonstrates for the first time that IL-22 and ILC3s can prevent excess inflammation and clear *Mtb* infection in T2DM and TB comorbid conditions, suggesting that IL-22 is a novel target for treating TB patients with T2DM.

IL-22 prevents experimental lung fibrosis, airway inflammation and tissue damage [56–59]. Previously, we found that *Mtb* infection induces severe immunopathology in the lungs of T2DM mice [19]. Furthermore, in the current study, we found a disintegrated epithelial barrier and loss of IL-22R expression by lung epithelial cells in T2DM mice infected with *Mtb*. Recombinant IL-22 treatment and ILC3 transfer prevented epithelial cell destruction, restored IL-22R expression, and resolved alveolar wall inflammation, which resulted in reduced alveolar destruction and fewer lesions in the lungs of T2DM mice infected with *Mtb*. We also found that IL-22 acted on lung epithelial cells to induce the expression of the anti-apoptotic proteins Bcl2 and Mcl1 and enhance antimicrobial peptide production by lung epithelial cells in T2DM mice infected with *Mtb* (Fig 4).

IL-22 can prevent pathogenic epithelial cell-destructive inflammation by inhibiting the release of matrix metalloproteases and PMN-recruiting chemokines and by promoting aberrant epithelial cell proliferation and differentiation [60–62]. We found an inverse correlation of IL-22 production with the level of elastase level and number of Ly6G+ cells in the lungs of the *Mtb*-infected T2DM mice. We found infiltrating Ly6G+ cells near the lung epithelial cell lining in *Mtb*-infected T2DM mice. Recombinant IL-22 treatment and ILC3 transfer had no effect on the total number of lung Ly6G+ cells but significantly reduced the infiltration of Ly6G+ cells near epithelial cells. Our results demonstrate that IL-22 prevents the accumulation of lung Ly6G+ cells near lung epithelial cells. Neutrophilic proteases can cleave IL-22R1 on epithelial cells and impair IL-22-dependent antimicrobial protein production from epithelial cells [63]. We found that neutrophils from T2DM mice infected with *Mtb* produced more neutrophil elastase 2 (ELA2) than nondiabetic mice infected with *Mtb*. Recombinant IL-22 treatment and ILC3 transfer decreased the production of neutrophil ELA2 in the lungs of *Mtb*-infected T2DM mice. ILC3s can also reduce CCL3 production and leukocyte recruitment at the site of injured tissue and ultimately inhibit inflammation [64,65]. Regulatory ILCs can also inhibit the generation of IL-17A/IFN-γ [66]. In addition to these findings, our study demonstrates for the first time that IL-22 and LTi cells prevent epithelial cell damage to inhibit infection-induced inflammation in a T2DM host. IL-22 is canonically associated with antimicrobial immunity [67]. IL-22 receptor signaling through STAT-3 in intestinal epithelial cells, induces the production of antimicrobial peptides [68]. Lung epithelial cell from IL-22 treated and *Mtb* infected T2DM mice had higher expression of the β-defensins, S100 calcium-binding proteins, and regenerating gene family (Reg) family proteins (Fig 4B). Our findings suggest that IL-22 treatment can control *Mtb* growth by inducing the production of anti-microbial proteins by lung epithelial cells in *Mtb* infected T2DM mice.

IL-22 is critical for the maintenance of the intestinal barrier function by promoting antipathogenic responses and the regeneration of epithelial cells [50,69]. We found that T2DM enhanced gut leakage in T2DM mice infected with *Mtb* and that recombinant IL-22 treatment prevented this permeability and maintained the integrity of the intestinal barrier (Fig 6). Our studies demonstrate that IL-22 not only prevents lung inflammation but also prevents gut leakage and maintains the integrity of the intestinal barrier to prevent other infections, the entry of dietary antigens into the circulation, pancreatic beta cell damage and insulin resistance in T2DM mice infected with *Mtb*. This defect in the lung enhances the susceptibility to *Mtb* infection, and in the colon, it allows bacteria to penetrate and induce systemic inflammation [43,70]. In addition, rIL-22 treatment and ILC3s adoptive transfer maintained glucose homeostasis and reduced dyslipidemia in *Mtb* infected T2DM mice. Our study demonstrates that IL-22 and ILC3s play an important role in mucosal defense and controlling the metabolic disorder in T2DM mice infected with *Mtb*.

In summary, our study demonstrates that IL-22 and ILC3s can prevent excess inflammation, reduce bacterial burden, improve lipid metabolism and prolong the survival of T2DM mice infected with *Mtb*. Further understanding of these mechanisms and human studies can help to treat TB patients with T2DM.

## Supporting information

S1 Fig IL-22 producing CD4, CD8, NK and γδ T cells in control and T2DM mice during *Mtb* infection. Control C57BL/6 and T2DM mice were infected with *Mtb* as shown in Fig 1 and described in the methods section. One, three and five months after *Mtb* infection, the absolute numbers of (A) CD4+, (B) CD8, (C) NK cells and (D) γδ T cells per lung were determined by flow cytometry. Five mice per group were used. The mean values, SDs and p-values are shown.
(TIF)

S2 Fig Gating strategy for the identification of ILC1s in mouse lung. Control C57BL/6 mice were infected with *Mtb* as shown in Fig 1 and described in the methods section. One, three and five post *Mtb* infection lung single cell suspension was prepared and flow cytometry was performed. Flow gating strategy for ILC1s (CD45+CD127+lin-NKp46+NK1.1+) are shown.
(TIF)

S3 Fig Gating strategy for the identification of IL-22 producing ILC1s and ILCs 2 in mouse lung. Control C57BL/6 mice were infected with *Mtb* as shown in Fig 1 and described in the methods section. One, three and five post *Mtb* infection lung single cell suspension was prepared and flow cytometry was performed. (A) Flow gating strategy for IL-22 and IFN-γ producing ILC1s (CD45+CD127+lin-NKp46+NK1.1+) and (B) IL-22 producing ILC2s (CD45+CD127+lin-Rorγt-Sca1+) are shown.
(TIF)

S4 Fig Interferon-gamma (IFN-γ)-producing type 1 innate lymphoid cells (ILC1s) in control and T2DM mice during *Mtb* infection. Control C57BL/6 and T2DM mice were infected with *Mtb* as shown in Fig 1 and described in the methods section. (A-D) One, three and five months after *Mtb* infection, the absolute number of ILC1 (CD45+CD127+lin-NKp46+NK1.1+) IFN-γ+ cells per 10^6^ cells in (A), lung, (B) spleen, (C), inguinal lymph nodes and (D) liver was determined by flow cytometry. Five mice per group were used. The mean values, SDs and p-values are shown.
(TIF)

S5 Fig Type 2 innate lymphoid cells (ILC2s) in control and T2DM mice during Mtb infection. Control C57BL/6 and T2DM mice were infected with *Mtb* as shown in Fig 1 and described in the methods section. (A-B) One, three and five months after *Mtb* infection, the absolute number of ILC2s (CD45+CD127+lin-Rorγt-Sca1+) per 10^6^ cells in (A) spleen and (B) lung was determined by flow cytometry. Five mice per group were used. The mean values, SDs and p-values are shown.
(TIF)

S6 Fig Gating strategy for the identification of ILC2s and ILC3s in mouse lung. Control C57BL/6 and T2DM mice were infected with *Mtb* as shown in Fig 1 and described in the methods section. One, three and five post *Mtb* infection lung single cell suspension were prepared and flow cytometry was performed. Flow gating strategies for ILC2s (CD45+CD127+lin-Rorγt-Sca1+) and ILC3s subpopulation LTi (CD45+CD127+lin-NK1.1-Rorγt+NKp46-CCR6+) and NCR+ (CD45+CD127+lin-NK1.1-Rorγt+NKp46+CCR6-) are shown.
(TIF)

S7 Fig IL-22 producing subpopulation of ILC3s. Control C57BL/6 and T2DM mice were infected with *Mtb* as shown in Fig 1 and described in the methods section. One, three and five months post *Mtb* infection lung single cell suspension was prepared and flowcytometry was performed. A representative flow cytometry figure for IL-22 producing (A) LTi and (B) NCR+ ILC3s is shown.
(TIF)

S8 Fig Recombinant-IL-22 treatment prolongs the survival of *Mtb*-infected T2DM mice. One month after the induction of diabetes, T2DM mice were infected with ~100 CFU of aerosolized *Mtb*. Five months after *Mtb* infection, mice were treated intravenously with recombinant IL-22 (100 ng/kg body weight, single dose) or PBS. (A) Schematic representation of *Mtb* infection and recombinant IL-22 treatment in T2DM mice is shown. (B) Survival of *Mtb*-infected T2DM mice treated with recombinant IL-22 or PBS. Survival curves were compared using the log rank test. Data were pooled from two independent experiments (n = 5 mice per group per experiment used). (C) Random blood glucose sampling at twenty-day intervals for up to 8 months. Five mice per group were used. The mean values, SDs and p-values are shown.
(TIF)

S9 Fig Adoptive transfer of type 3 innate lymphoid cells (ILC3s) enhances the survival of *Mtb*-infected T2DM mice. T2DM (CD45.2, C57BL/6 background) mice were infected with ~100 CFU of aerosolized *Mtb*. Five months after *Mtb* infection, 0.5 x 10^5^ NCR+ (Lin-CD127+NK1.1-NKp46+CCR6-) or LTi+ (Lin-CD127+NK1.1-NKp46-CCR6+) pooled cells (from spleen, lung, liver, lymph nodes and mucosal sites) from CD45.1 mice (C57BL/6) were adoptively transferred via tail vein injection (recipient CD45.2 *Mtb*-infected T2DM mice). Fifteen and thirty days after adoptive transfer, CD45.1+ILC3+ cells were analyzed in the (A) lung, (B) spleen and (C) liver by flow cytometry.
(TIF)

S10 Fig IL-22 levels in the lungs after adoptive transfer of ILC3s. T2DM (CD45.2, C57BL/6 background) mice were infected with ~100 CFU of aerosolized *Mtb*. Five months after *Mtb* infection, NCR+ (Lin-CD127+NK1.1-NKp46+CCR6-) or LTi+ (Lin-CD127+NK1.1-NKp46-CCR6+) cells were isolated from pooled spleen, lung, liver, lymph nodes of CD45.1 mice (C57BL/6). 0.5 x 10^5^ NCR+ (Lin-CD127+NK1.1-NKp46+CCR6-) or LTi+ (Lin-CD127+NK1.1-NKp46-CCR6+) cells were adoptively transferred to CD45.2 *Mtb*-infected T2DM mice (recipient) via tail vein injection. Thirty days after adoptive transfer, the level of IL-22 was measured in the (A) lung homogenate and (B) plasma of recipient mice by ELISA. Five mice per group were used. The mean values, SDs and p-values are shown.
(TIF)

S11 Fig Accumulation of phagocytic cells near the alveolar epithelial cell lining in recombinant IL-22-treated *Mtb*-infected T2DM mice. One month after the induction of diabetes, T2DM mice were infected with ~100 CFU of aerosolized *Mtb* as shown in Fig 1 and described in the methods section. Five months after *Mtb* infection, T2DM mice were treated intravenously with either recombinant IL-22 (100 ng/kg body weight, twice weekly) or PBS. (A) After one month of recombinant IL-22 treatment, the lungs were isolated and formalin fixed. Paraffin-embedded tissue sections were prepared, and immunofluorescence staining was performed. Stained tissue sections were analyzed by confocal microscopy to determine the accumulation of F4/80+ (magenta) and CD11C+ (red) cells near EpCAM+ cells (green). (B) Paraffin-embedded tissue sections were analyzed by confocal microscopy to determine the accumulation of Ly6G+ cells (magenta) near the alveolar epithelial cell lining (green).
(TIF)

S12 Fig Level of myeloperoxidase (MPO) and elastase 2 in the lung homogenate of control and T2DM mice during *Mtb* infection. Control C57BL/6 and T2DM mice were infected with *Mtb* as shown in Fig 1 and described in the methods section. Five months after *Mtb* infection, (A) MPO and (B) elastase levels were measured in lung homogenates by ELISA. (C) The frequency of the Ly6G+ cells was measured by flow cytometry. Five mice per group were used. The mean values, SDs and p-values are shown.
(TIF)

S13 Fig IL-22 treatment maintains gut epithelial cell integrity in *Mtb*-infected T2DM mice. Control and T2DM mice were infected with 50–100 CFU of aerosolized *Mtb*. Five months after *Mtb* infection, gut permeability was determined by the oral delivery of fluorescein isothiocyanate-dextran (FITC-dextran) (44 mg/100 gm body weight). (A) Six hours after oral delivery, the serum level of FITC-dextran was measured by fluorometry. (B) Five months after *Mtb* infection, T2DM mice were treated intravenously with either recombinant IL-22 (100 ng/kg body weight, twice weekly) or PBS. One month after recombinant IL-22 or PBS treatment, FITC-dextran was orally delivered, and after six hours, serum FITC-dextran level were measured. Five mice per group were used. The mean values, SDs and p-values are shown. (C) After one month of recombinant IL-22 treatment, the small intestine was isolated and formalin fixed. Paraffin-embedded intestinal tissue sections were prepared, and hematoxylin and eosin staining was performed. Representative hematoxylin- and eosin-stained small intestine tissue sections are shown. Five mice per group were used.
(TIF)

S1 Table Demographic details. (DOCX)

S2 Table List of primers used in this study. (DOCX)
