# Correction: IL-22 produced by type 3 innate lymphoid cells (ILC3s) reduces the mortality of type 2 diabetes mellitus (T2DM) mice infected with *Mycobacterium tuberculosis*

**DOI:** 10.1371/journal.ppat.1009578

**Published:** 2021-05-06

**Authors:** Deepak Tripathi, Rajesh Kumar Radhakrishnan, Ramya Sivangala Thandi, Padmaja Paidipally, Kamakshi Prudhula Devalraju, Venkata Sanjeev Kumar Neela, Madeline Kay McAllister, Buka Samten, Vijaya Lakshmi Valluri, Ramakrishna Vankayalapati

In Fig 6B, the boxes in 40X histology images are incorrectly positioned to correspond to the 100X images. Please see the correct Fig 6 below.

**Fig 6:**
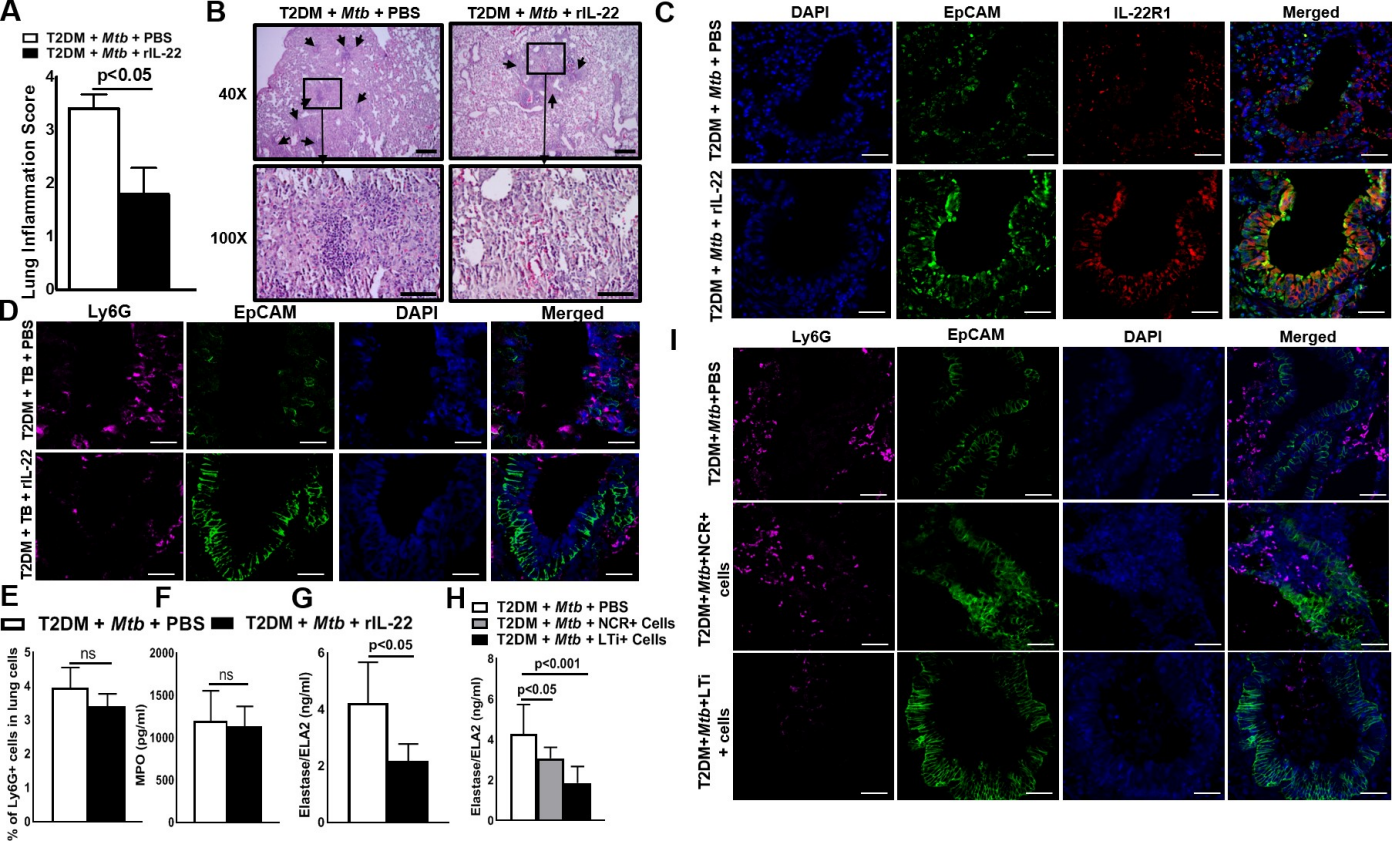
IL-22 reduces the severity of lung inflammation and neutrophil-mediated damage of lung epithelial cells in *Mtb*-infected T2DM mice. One month after the induction of diabetes, T2DM mice were infected with ~100 CFU of aerosolized *Mtb* as shown in Fig 1 and described in the methods section. Five months after *Mtb* infection, T2DM mice were treated intravenously with either recombinant IL-22 (100 ng/kg body weight, twice weekly) or PBS. (A) After one month of recombinant IL-22 treatment, the lungs were isolated and formalin fixed. Paraffin-embedded tissue sections were prepared, and hematoxylin and eosin staining was performed. Lung inflammation scores were quantified by morphometric analysis. (B) Representative hematoxylin and eosin-stained lung tissue sections in multiple fields (at 40X and 100X) are shown. (C) Paraffin-embedded tissue sections were analyzed by confocal microscopy to determine the colocalization of EpCAM-positive cells (green) and IL-22R1+ cells (red). (D) Paraffin-embedded tissue sections were analyzed by confocal microscopy to determine the accumulation of the Ly6G+ cells (magenta) near the EpCAM+ epithelial cell lining (green). (E) The frequencies of Ly6G+ lung cell populations were determined by flow cytometry staining at 1 month after recombinant IL-22 treatment. (F-G) One month after PBS or recombinant IL-22 treatment, lung homogenates of the *Mtb*-infected T2DM mice were collected, and the levels of (F) myeloperoxidase (MPO) and (G) elastase were measured by ELISA. (H and I) T2DM (CD45.2, C57BL/6 background) mice were infected with 50–100 CFU of aerosolized *Mtb*. Five months after *Mtb* infection, 0.5 x 105 NCR+ or LTi+ pooled cells (from spleen, lung, liver, lymph nodes and mucosal sites) from CD45.1 mice (C57BL/6) were adoptively transferred via tail vein injection (recipient CD45.2 Mtb-infected T2DM mice). (H) Elastase levels in the lung homogenate were measured by ELISA. (I) Ly6G+ cell accumulation near EpCAM+ cells (epithelial cell lining) in the lungs of ILC3- or PBS-treated Mtb-infected T2DM mice was analyzed by confocal microscopy. A representative immunofluorescence image is shown. Five mice per group were used. The mean values, SDs and p-values are shown.

In S7A and S7B Fig, the incorrect representative flow cytometry images were used for the 5-month T2DM + *Mtb* group. Please see the correct S7 Fig below.

## Supporting information

S7 Fig IL-22 producing subpopulation of ILC3s. Control C57BL/6 and T2DM mice were infected with *Mtb* as shown in Fig 1 and described in the methods section. One, three and five months post *Mtb* infection lung single cell suspension was prepared and flowcytometry was performed. A representative flow cytometry figure for IL-22 producing (A) LTi and (B) NCR+ ILC3s is shown.
(TIFF)
