# Supplementary figures and images for: IL-22 produced by type 3 innate lymphoid cells (ILC3s) reduces the mortality of type 2 diabetes mellitus (T2DM) mice infected with Mycobacterium tuberculosis
Source: PLoS Pathog. 2019 Dec 6;15(12):e1008140. doi: 10.1371/journal.ppat.1008140 (PMC6919622; doi:10.1371/journal.ppat.1008140)

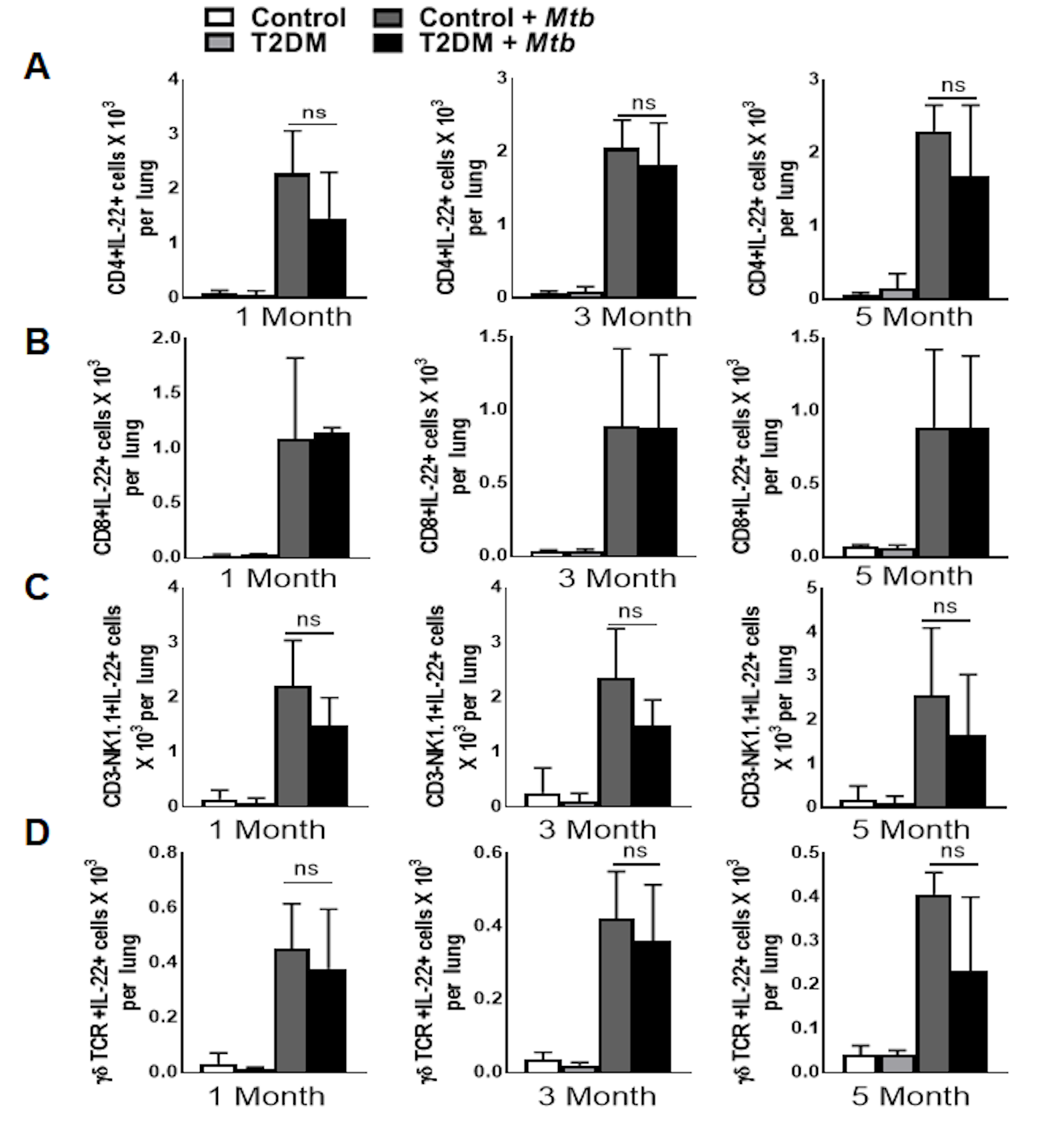

Supplement: S1 Fig — Control C57BL/6 and T2DM mice were infected with Mtb as shown in Fig 1 and described in the methods section. One, three and five months after Mtb infection, the absolute numbers of (A) CD4+, (B) CD8, (C) NK cells and (D) γδ T cells per lung were determined by flow cytometry. Five mice per group were used. The mean values, SDs and p-values are shown. (TIF) [file ppat.1008140.s001.tif]

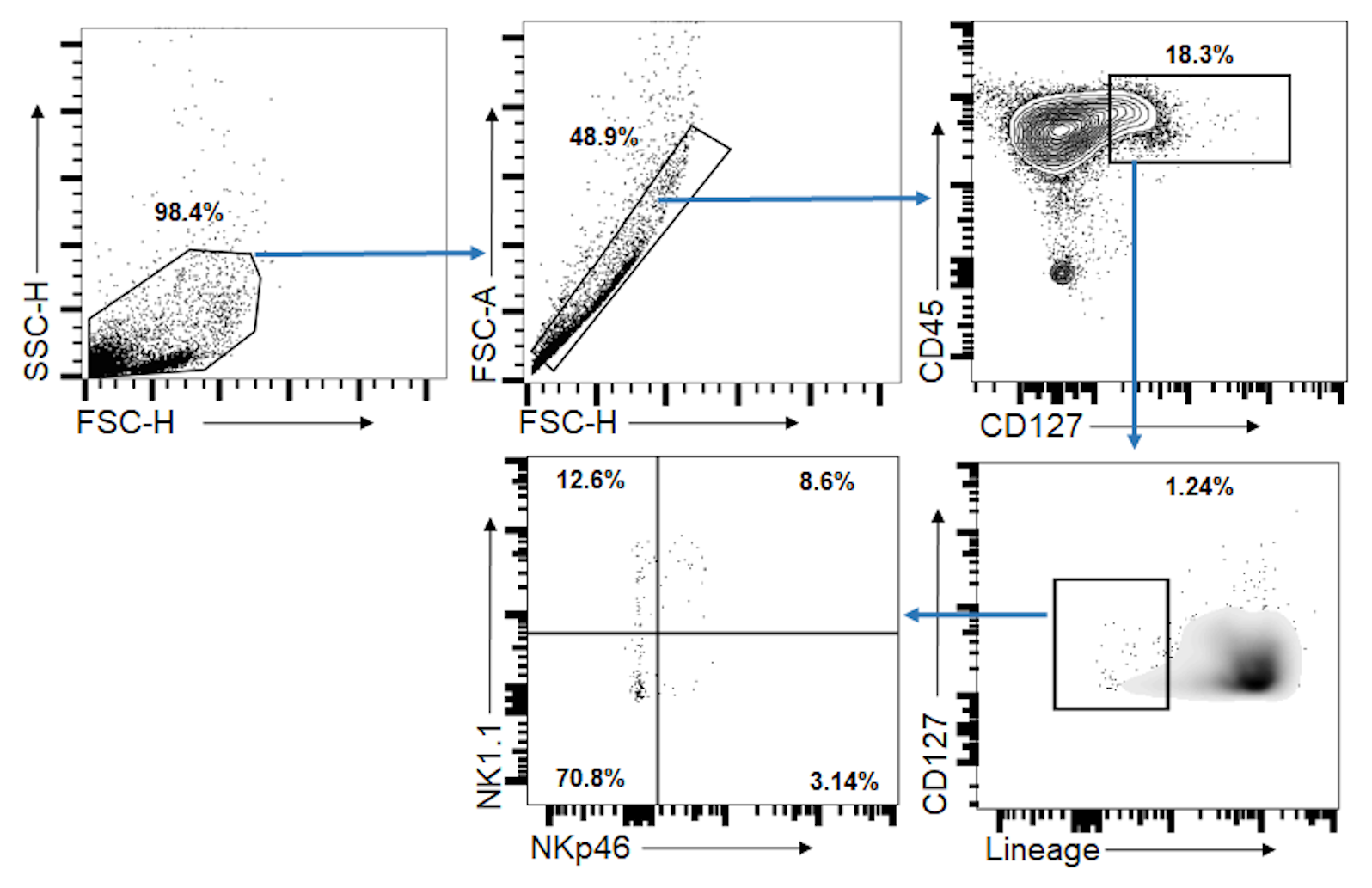

Supplement: S2 Fig — Control C57BL/6 mice were infected with Mtb as shown in Fig 1 and described in the methods section. One, three and five post Mtb infection lung single cell suspension was prepared and flow cytometry was performed. Flow gating strategy for ILC1s (CD45+CD127+lin-NKp46+NK1.1+) are shown. (TIF) [file ppat.1008140.s002.tif]

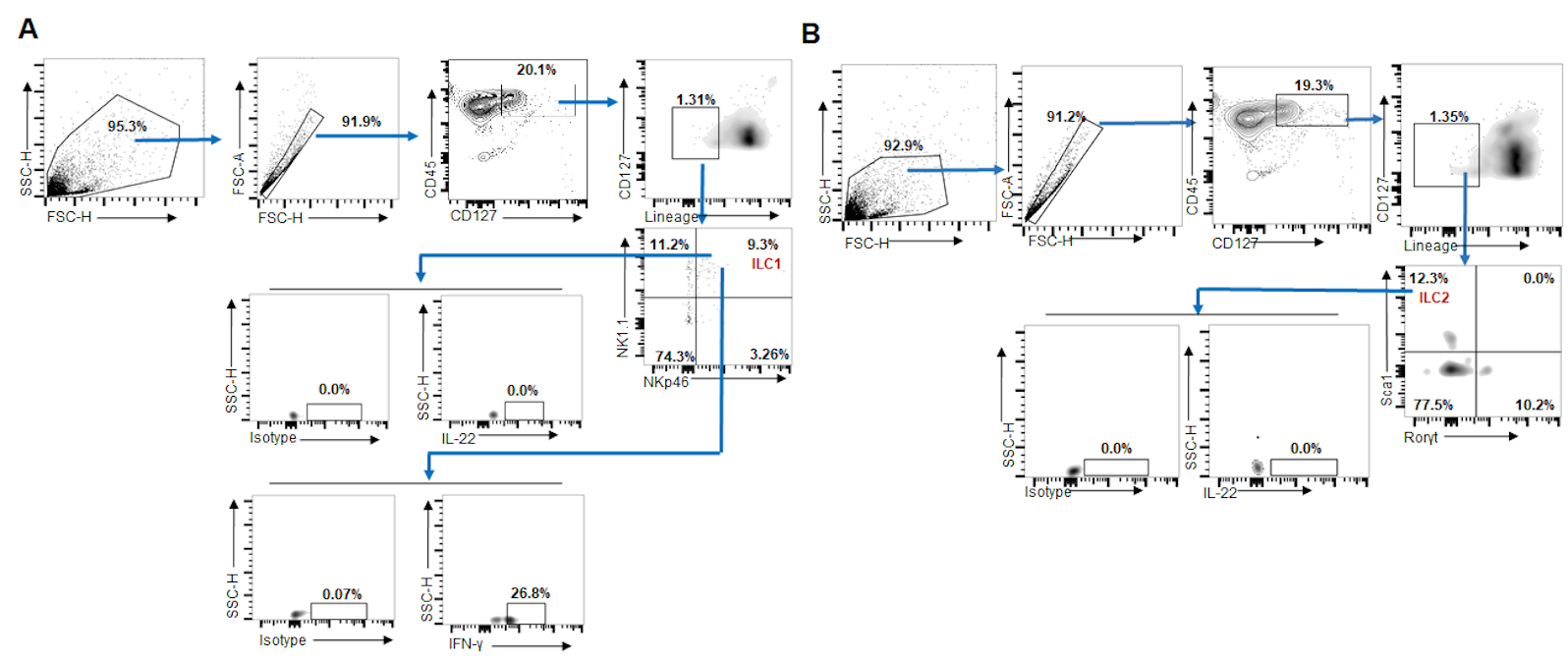

Supplement: S3 Fig — Control C57BL/6 mice were infected with Mtb as shown in Fig 1 and described in the methods section. One, three and five post Mtb infection lung single cell suspension was prepared and flow cytometry was performed. (A) Flow gating strategy for IL-22 and IFN-γ producing ILC1s (CD45+CD127+lin-NKp46+NK1.1+) and (B) IL-22 producing ILC2s (CD45+CD127+lin-Rorγt-Sca1+) are shown. (TIF) [file ppat.1008140.s003.tif]

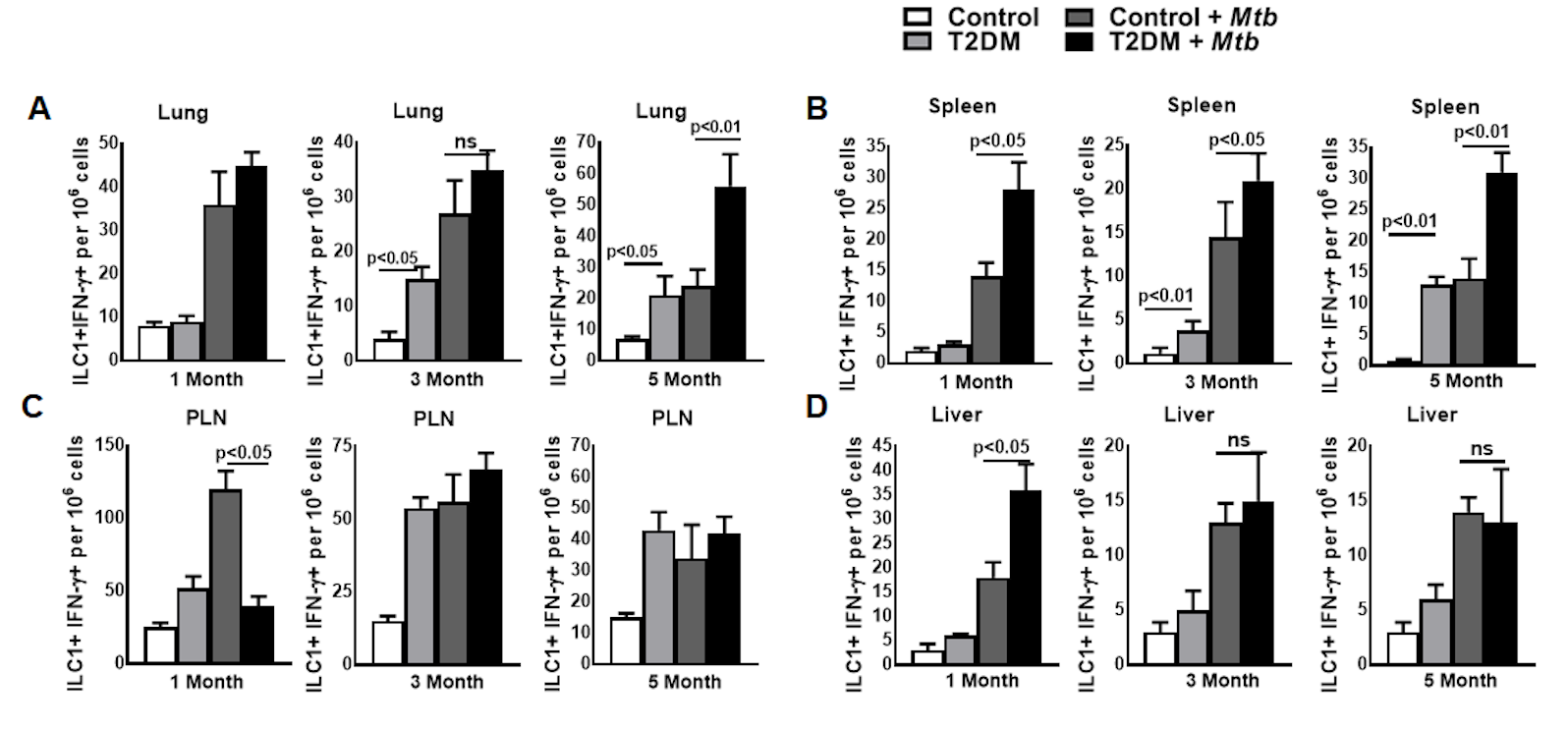

Supplement: S4 Fig — Control C57BL/6 and T2DM mice were infected with Mtb as shown in Fig 1 and described in the methods section. (A-D) One, three and five months after Mtb infection, the absolute number of ILC1 (CD45+CD127+lin-NKp46+NK1.1+) IFN-γ+ cells per 106 cells in (A), lung, (B) spleen, (C), inguinal lymph nodes and (D) liver was determined by flow cytometry. Five mice per group were used. The mean values, SDs and p-values are shown. (TIF) [file ppat.1008140.s004.tif]

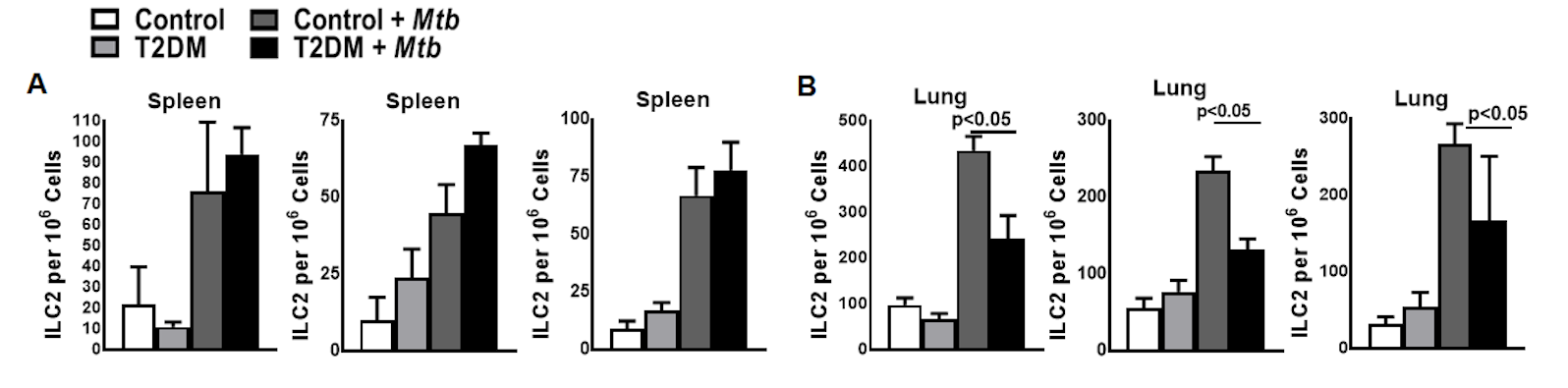

Supplement: S5 Fig — Control C57BL/6 and T2DM mice were infected with Mtb as shown in Fig 1 and described in the methods section. (A-B) One, three and five months after Mtb infection, the absolute number of ILC2s (CD45+CD127+lin-Rorγt-Sca1+) per 106 cells in (A) spleen and (B) lung was determined by flow cytometry. Five mice per group were used. The mean values, SDs and p-values are shown. (TIF) [file ppat.1008140.s005.tif]

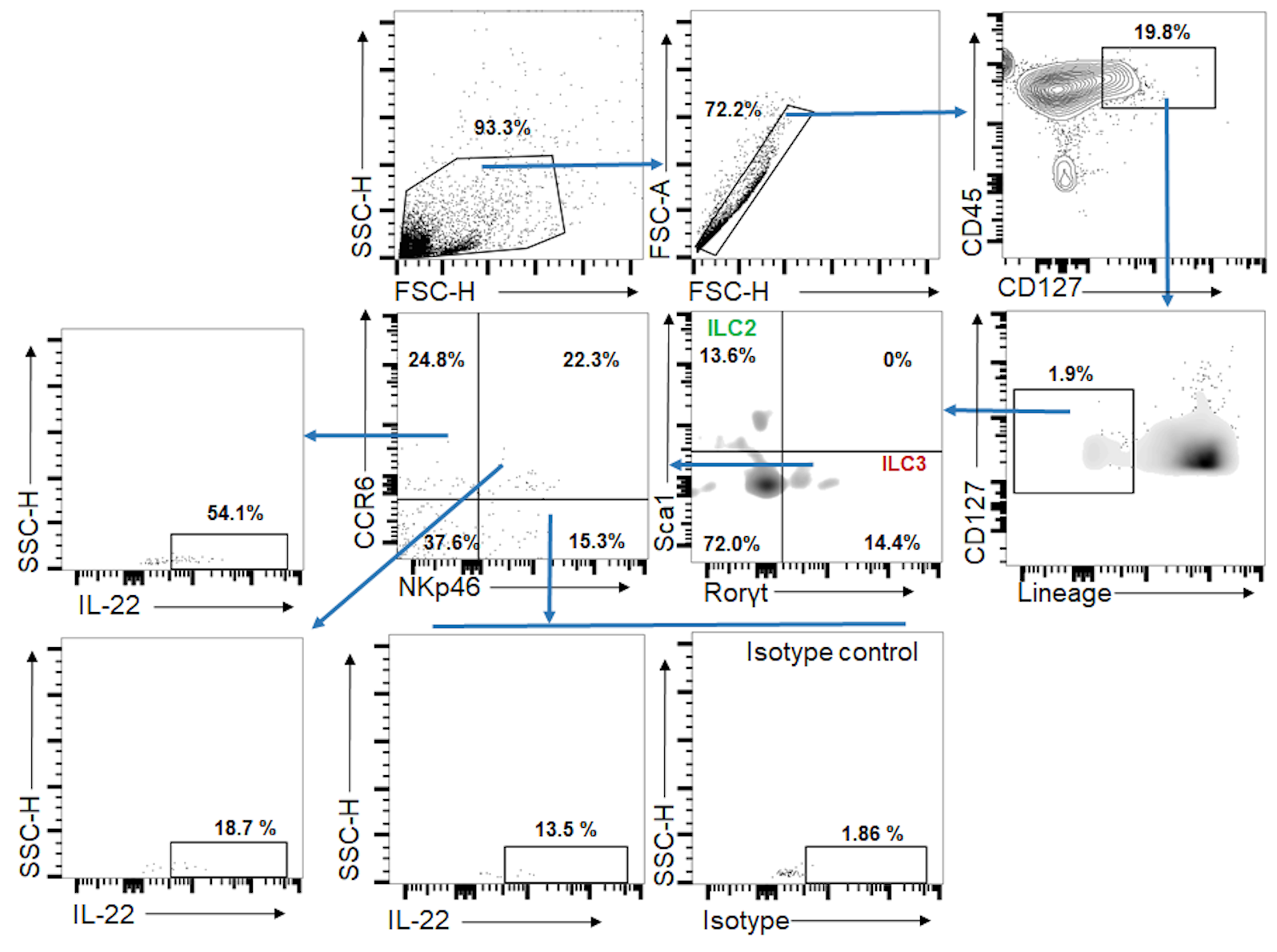

Supplement: S6 Fig — Control C57BL/6 and T2DM mice were infected with Mtb as shown in Fig 1 and described in the methods section. One, three and five post Mtb infection lung single cell suspension were prepared and flow cytometry was performed. Flow gating strategies for ILC2s (CD45+CD127+lin-Rorγt-Sca1+) and ILC3s subpopulation LTi (CD45+CD127+lin-NK1.1-Rorγt+NKp46-CCR6+) and NCR+ (CD45+CD127+lin-NK1.1-Rorγt+NKp46+CCR6-) are shown. (TIF) [file ppat.1008140.s006.tif]

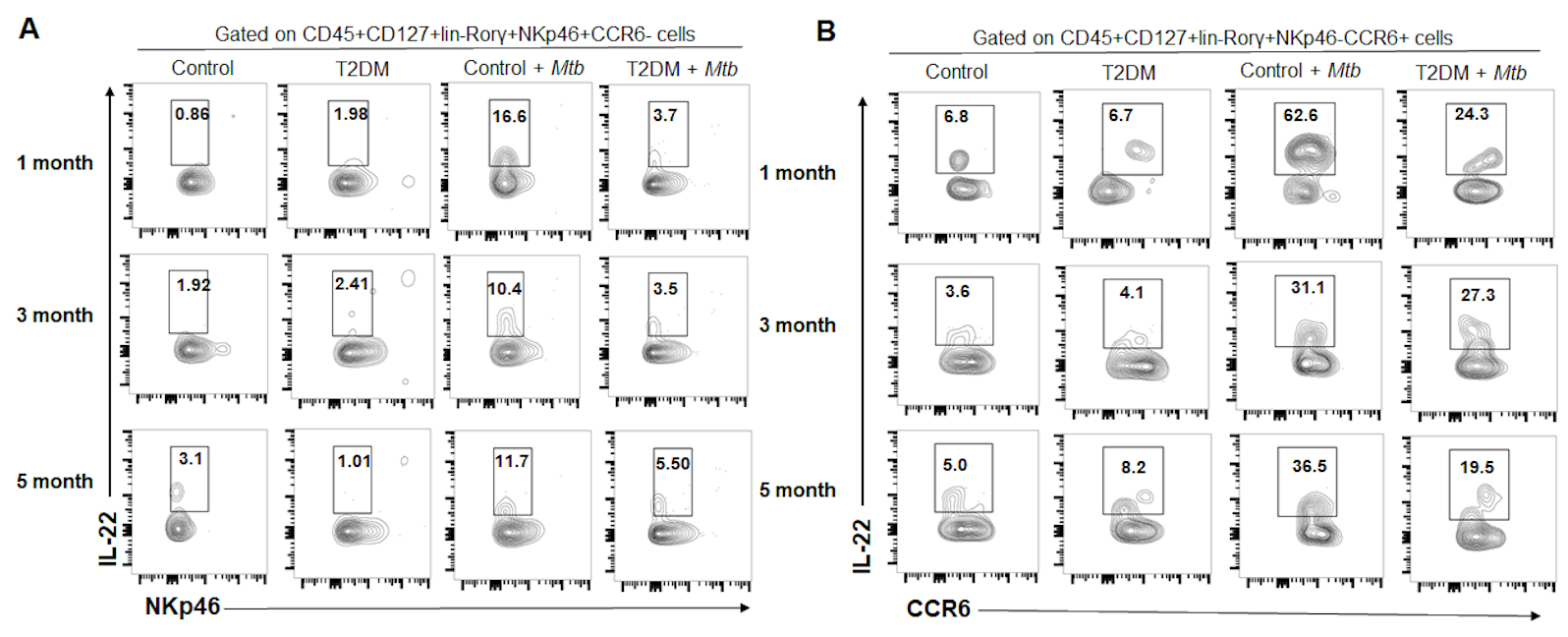

Supplement: S7 Fig — Control C57BL/6 and T2DM mice were infected with Mtb as shown in Fig 1 and described in the methods section. One, three and five months post Mtb infection lung single cell suspension was prepared and flowcytometry was performed. A representative flow cytometry figure for IL-22 producing (A) LTi and (B) NCR+ ILC3s is shown. (TIF) [file ppat.1008140.s007.tif]

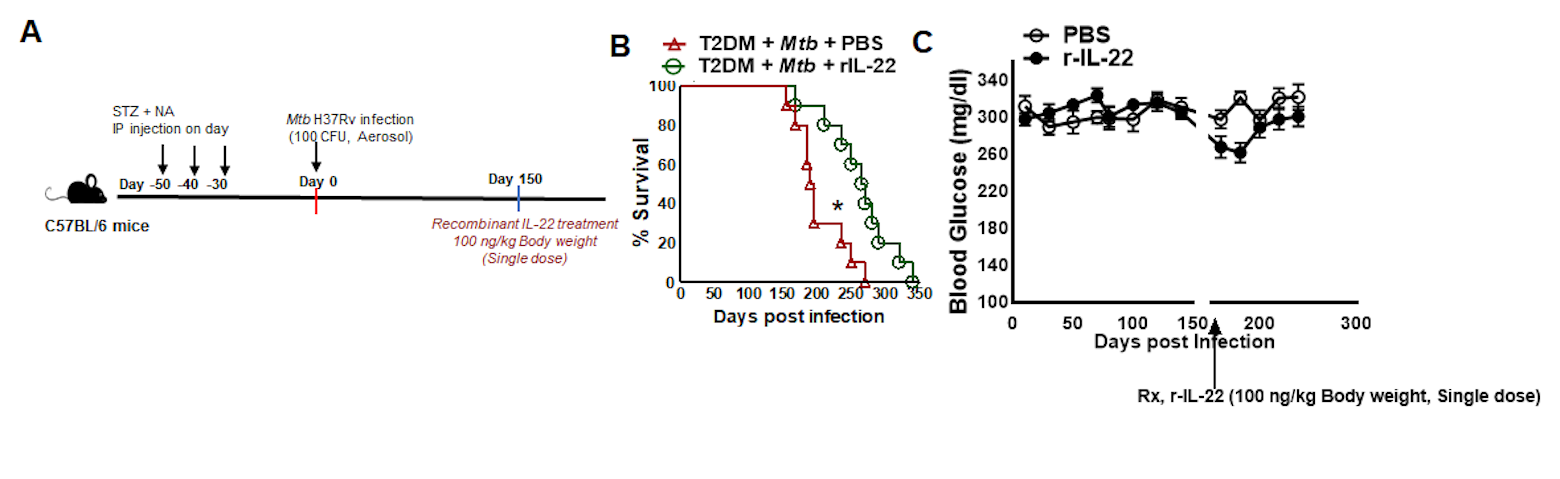

Supplement: S8 Fig — One month after the induction of diabetes, T2DM mice were infected with ~100 CFU of aerosolized Mtb. Five months after Mtb infection, mice were treated intravenously with recombinant IL-22 (100 ng/kg body weight, single dose) or PBS. (A) Schematic representation of Mtb infection and recombinant IL-22 treatment in T2DM mice is shown. (B) Survival of Mtb-infected T2DM mice treated with recombinant IL-22 or PBS. Survival curves were compared using the log rank test. Data were pooled from two independent experiments (n = 5 mice per group per experiment used). (C) Random blood glucose sampling at twenty-day intervals for up to 8 months. Five mice per group were used. The mean values, SDs and p-values are shown. (TIF) [file ppat.1008140.s008.tif]

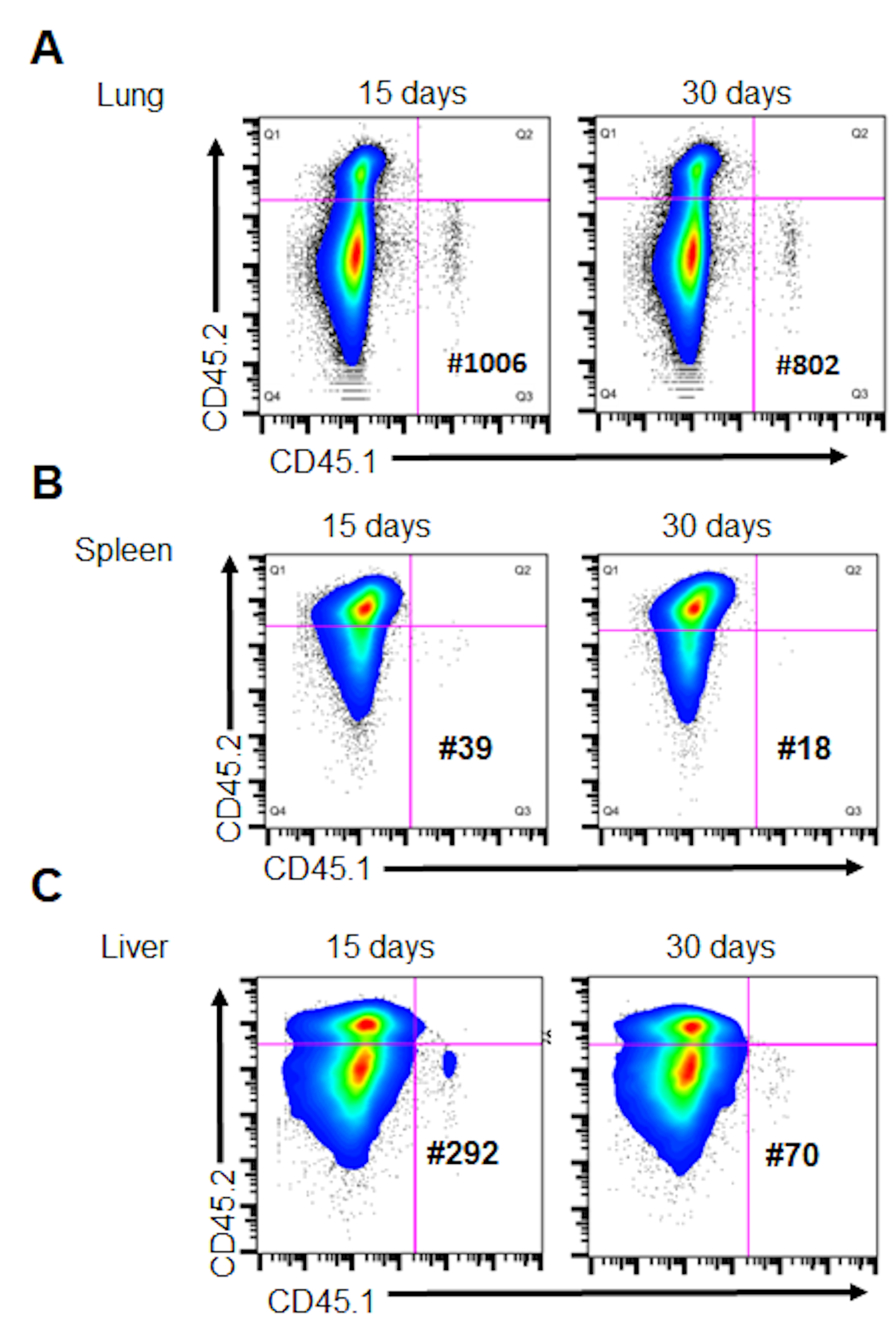

Supplement: S9 Fig — T2DM (CD45.2, C57BL/6 background) mice were infected with ~100 CFU of aerosolized Mtb. Five months after Mtb infection, 0.5 x 10^5^ NCR+ (Lin-CD127+NK1.1-NKp46+CCR6-) or LTi+ (Lin-CD127+NK1.1-NKp46-CCR6+) pooled cells (from spleen, lung, liver, lymph nodes and mucosal sites) from CD45.1 mice (C57BL/6) were adoptively transferred via tail vein injection (recipient CD45.2 Mtb-infected T2DM mice). Fifteen and thirty days after adoptive transfer, CD45.1+ILC3+ cells were analyzed in the (A) lung, (B) spleen and (C) liver by flow cytometry. (TIF) [file ppat.1008140.s009.tif]

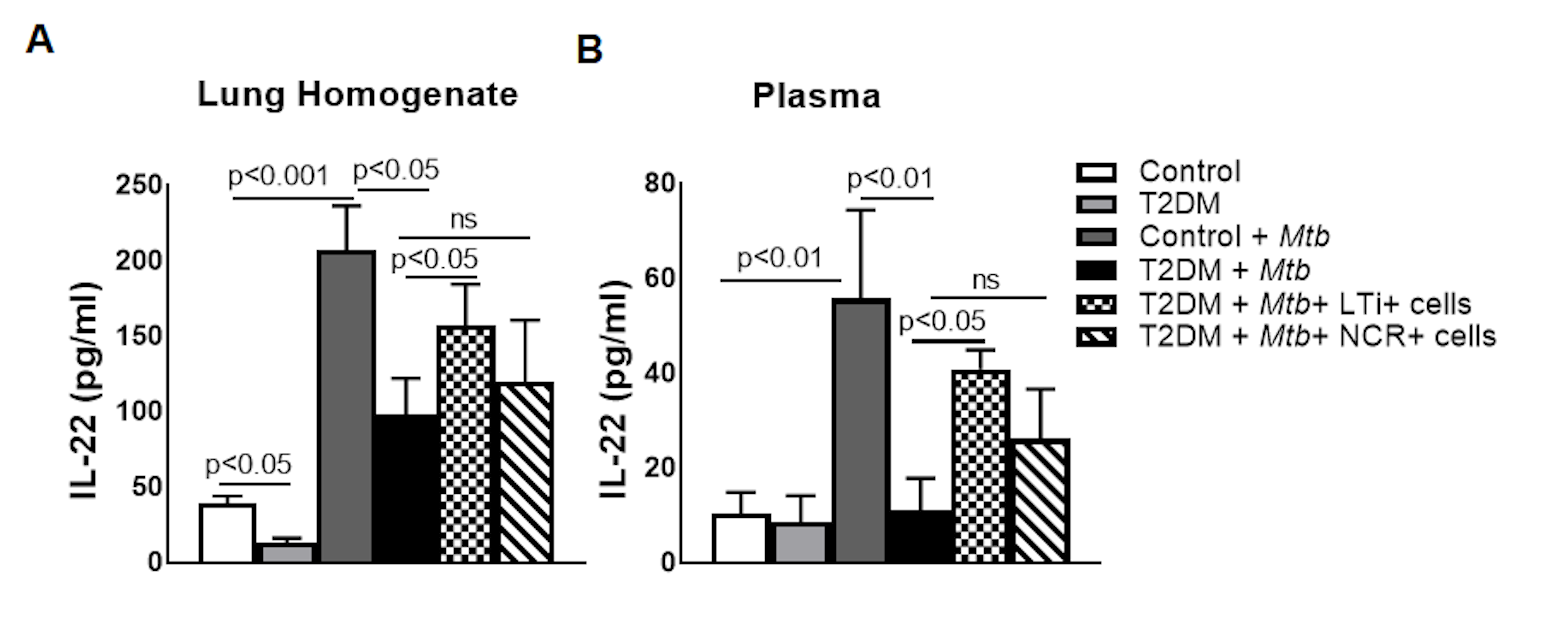

Supplement: S10 Fig — T2DM (CD45.2, C57BL/6 background) mice were infected with ~100 CFU of aerosolized Mtb. Five months after Mtb infection, NCR+ (Lin-CD127+NK1.1-NKp46+CCR6-) or LTi+ (Lin-CD127+NK1.1-NKp46-CCR6+) cells were isolated from pooled spleen, lung, liver, lymph nodes of CD45.1 mice (C57BL/6). 0.5 x 10^5^ NCR+ (Lin-CD127+NK1.1-NKp46+CCR6-) or LTi+ (Lin-CD127+NK1.1-NKp46-CCR6+) cells were adoptively transferred to CD45.2 Mtb-infected T2DM mice (recipient) via tail vein injection. Thirty days after adoptive transfer, the level of IL-22 was measured in the (A) lung homogenate and (B) plasma of recipient mice by ELISA. Five mice per group were used. The mean values, SDs and p-values are shown. (TIF) [file ppat.1008140.s010.tif]

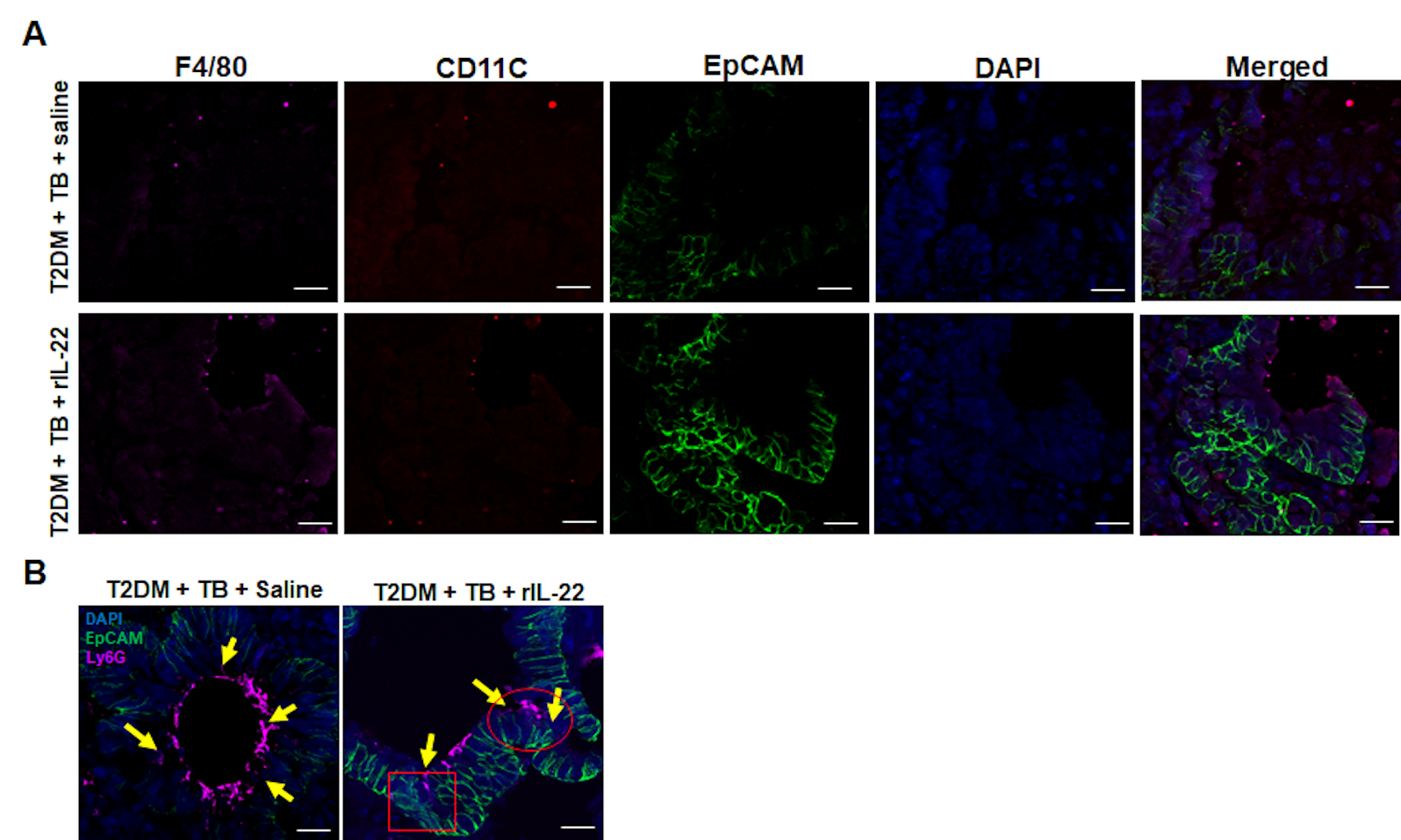

Supplement: S11 Fig — One month after the induction of diabetes, T2DM mice were infected with ~100 CFU of aerosolized Mtb as shown in Fig 1 and described in the methods section. Five months after Mtb infection, T2DM mice were treated intravenously with either recombinant IL-22 (100 ng/kg body weight, twice weekly) or PBS. (A) After one month of recombinant IL-22 treatment, the lungs were isolated and formalin fixed. Paraffin-embedded tissue sections were prepared, and immunofluorescence staining was performed. Stained tissue sections were analyzed by confocal microscopy to determine the accumulation of F4/80+ (magenta) and CD11C+ (red) cells near EpCAM+ cells (green). (B) Paraffin-embedded tissue sections were analyzed by confocal microscopy to determine the accumulation of Ly6G+ cells (magenta) near the alveolar epithelial cell lining (green). (TIF) [file ppat.1008140.s011.tif]

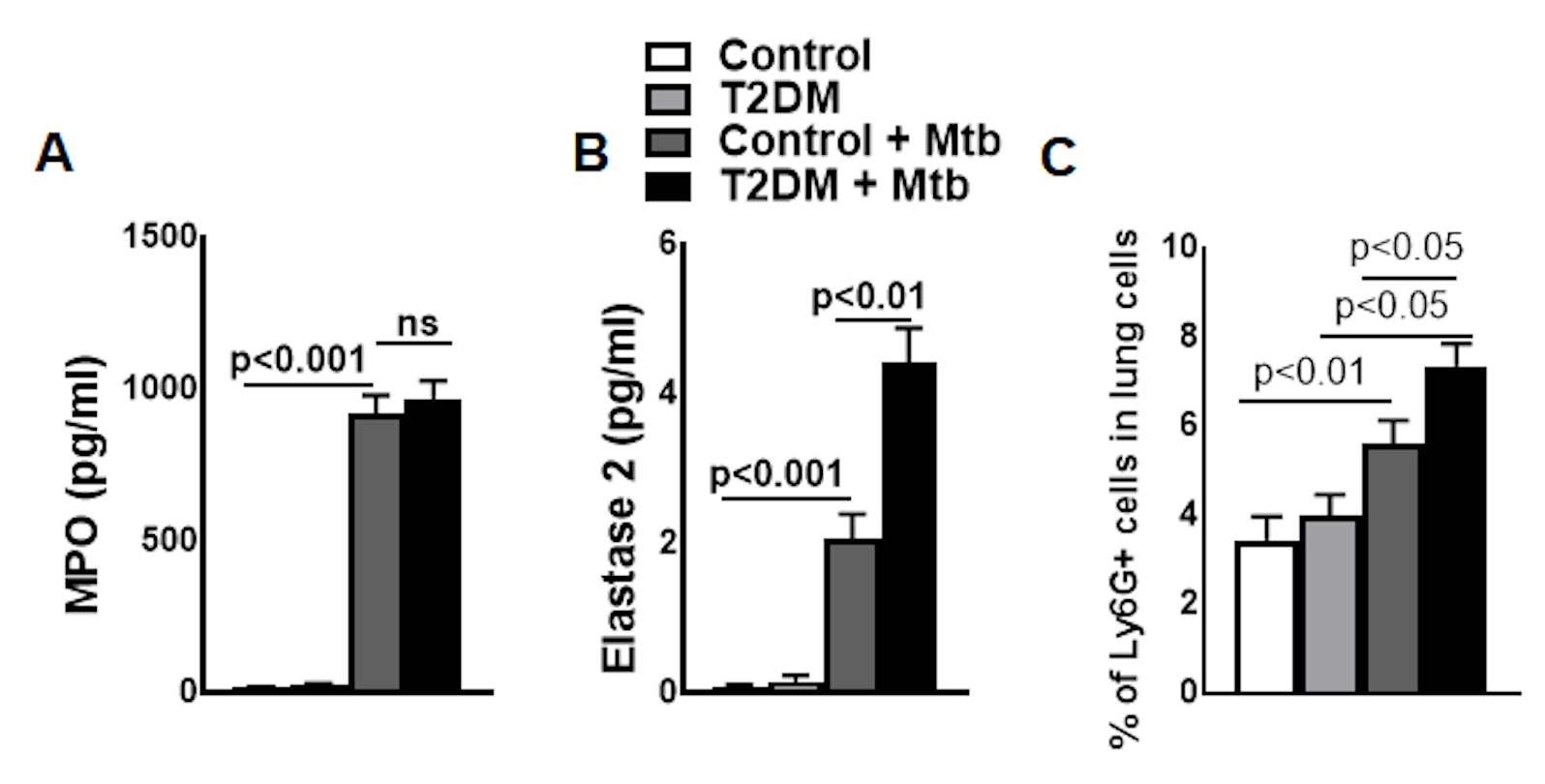

Supplement: S12 Fig — Control C57BL/6 and T2DM mice were infected with Mtb as shown in Fig 1 and described in the methods section. Five months after Mtb infection, (A) MPO and (B) elastase levels were measured in lung homogenates by ELISA. (C) The frequency of the Ly6G+ cells was measured by flow cytometry. Five mice per group were used. The mean values, SDs and p-values are shown. (TIF) [file ppat.1008140.s012.tif]

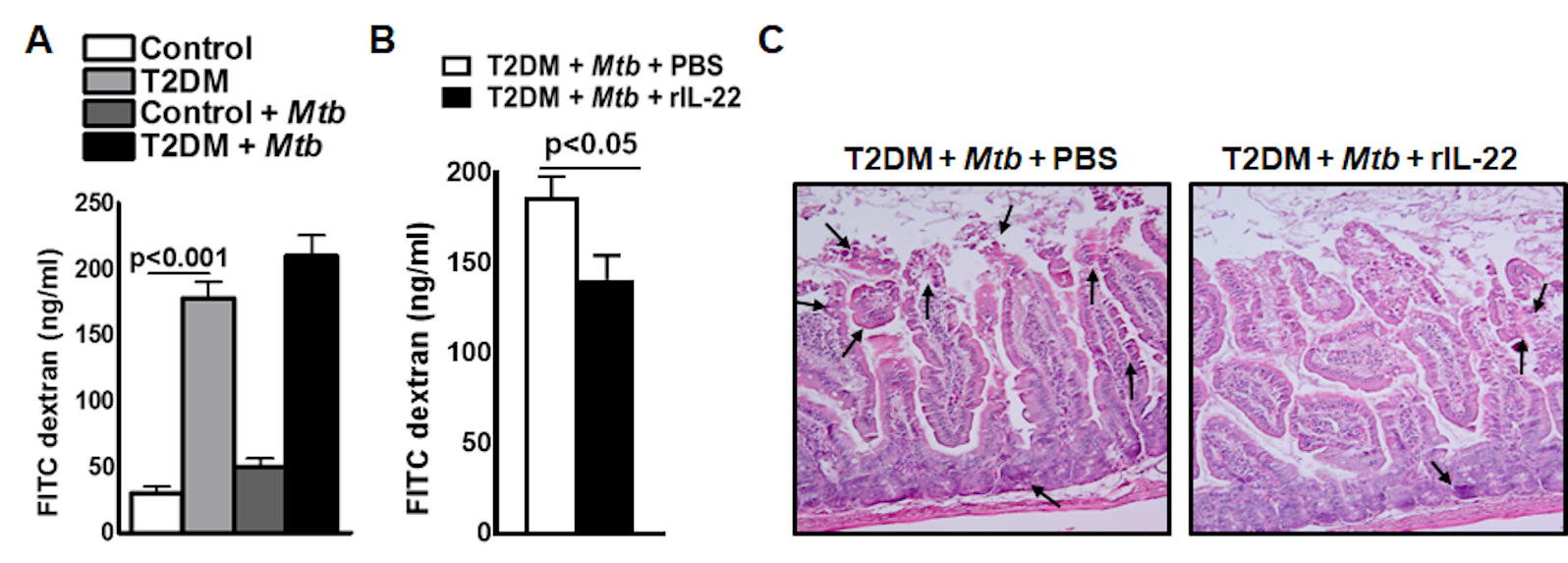

Supplement: S13 Fig — Control and T2DM mice were infected with 50–100 CFU of aerosolized Mtb. Five months after Mtb infection, gut permeability was determined by the oral delivery of fluorescein isothiocyanate-dextran (FITC-dextran) (44 mg/100 gm body weight). (A) Six hours after oral delivery, the serum level of FITC-dextran was measured by fluorometry. (B) Five months after Mtb infection, T2DM mice were treated intravenously with either recombinant IL-22 (100 ng/kg body weight, twice weekly) or PBS. One month after recombinant IL-22 or PBS treatment, FITC-dextran was orally delivered, and after six hours, serum FITC-dextran level were measured. Five mice per group were used. The mean values, SDs and p-values are shown. (C) After one month of recombinant IL-22 treatment, the small intestine was isolated and formalin fixed. Paraffin-embedded intestinal tissue sections were prepared, and hematoxylin and eosin staining was performed. Representative hematoxylin- and eosin-stained small intestine tissue sections are shown. Five mice per group were used. (TIF) [file ppat.1008140.s013.tif]
