# Supplementary material for: IL-22 produced by type 3 innate lymphoid cells (ILC3s) reduces the mortality of type 2 diabetes mellitus (T2DM) mice infected with Mycobacterium tuberculosis
Source: PLoS Pathog. 2019 Dec 6;15(12):e1008140. doi: 10.1371/journal.ppat.1008140 (PMC6919622; doi:10.1371/journal.ppat.1008140)
Supplement: S1 Table — (DOCX) [file ppat.1008140.s014.docx]

| **Demographic details of study population** | | |
| --- | --- | --- |
|  | TB (*N = 32*) | TB + T2DM (*N = 14*) |
| Gender (Male : Female) | 12:20 | 06:08 |
| Age (Range) | (18-43) | (30-55) |
| HIV status | 0% | 0% |
| TB status | 100% | 100% |
| BCG Vaccination status | 26 Yes, 6 Unknown | 8 Yes, 6 Unknown |
|  | | Average years of T2DM onset: 4.21 years |
|  |  | Most recent average Fasting blood glucose level: 135 mg/dl |
|  |  | Most recent average post prandial blood sugar: 230 mg/dl. |

**Supplementary Table 1: Demographic details**

**Supplementary Table 1:**
