# Supplementary material for: IL-22 produced by type 3 innate lymphoid cells (ILC3s) reduces the mortality of type 2 diabetes mellitus (T2DM) mice infected with Mycobacterium tuberculosis
Source: PLoS Pathog. 2019 Dec 6;15(12):e1008140. doi: 10.1371/journal.ppat.1008140 (PMC6919622; doi:10.1371/journal.ppat.1008140)
Supplement: S2 Table — (DOCX) [file ppat.1008140.s015.docx]

| **S.No**  **Supplementary Table 2: List of primers used in this study** | **Gene Name** | **Mouse Primer Sequences** |
| --- | --- | --- |
| 1. | CRAMP | AAGGAA-CAGGGGGTGGTG |
|  |  | CCGGGAAATTTTCTTGAACC |
| 2. | PGLA2 | GGC CTT TGG CTC AAT ACA GGT C |
|  |  | ACA GTG GCA TCC ATA GAA GGC |
| 3. | MMP7 | AGA TGT GGA GTG CCA GAT GT |
|  |  | TAG ACT GCT ACC ATC CGT CC |
| 4. | Reg3b | AAGAATATACCCTCCGCACGC |
|  |  | CAGACATAGGGCAACTTCACC |
| 5. | Reg3g | GCTCCTATTGCTATGCCTTGTTTAG |
|  |  | CATGGAGGACAGGAAGGAAGC |
| 6. | BPI | TAATAGCTAGCATGACCTGGGCCCCTGACA |
|  |  | GCCGCCTCGAGTTAGATAAGGTGTAAATCCGCTTC |
| 7. | Defensin-a | AAGAGACTAAAACTGAGGAGCAGC |
|  |  | CGCAGCAGAGCGTGTA |
| 8. | Oasl12 | GGATGCCTGGGAGAGAATCG |
|  |  | TCGCCTGCTCTTCGAAACTG |
| 9. | INOS | GCA GAA TGT GAC CAT CAT GG |
|  |  | ACA ACC TTG GTG TTG AAG GC |
| 10. | NOS2 | GTCATGGCTTCACGGGTCAG |
|  |  | CCAGGTCCCTGGCTAGTGCT |
| 11. | MBD-1 | CCAGATGGAGCCAGGTGTTG |
|  |  | AGCTGGAGCGGAGACAGAATCC |
| 12. | MBD-2 | AAGTATTGGATACGAAGCAG |
|  |  | TGGCAGAAGGAGGACAAATG |
| 13. | MBD-3 | GCATTGGCAACACTCGTCAGA |
|  |  | CGGGATCTTGGTCTTCTCTA |
| 14. | MBD-4 | GCAGCCTTTACCCAAATTATC |
|  |  | ACAATTGCCAATCTGTCGAA |
| 15. | SP-D | TGTGATGGTGGGAATGGGTCAGAA |
|  |  | TGTGGTGCCAGATCTTCTCCATGT |
| 16. | β- actin | TTACAGGAAGTCCCTCACCC |
|  |  | ACACAGAAGCAATGCTGTCAC |
