# Supplementary figures and images for: Correction: IL-22 produced by type 3 innate lymphoid cells (ILC3s) reduces the mortality of type 2 diabetes mellitus (T2DM) mice infected with Mycobacterium tuberculosis
Source: PLoS Pathog. 2021 May 6;17(5):e1009578. doi: 10.1371/journal.ppat.1009578 (PMC8101912; doi:10.1371/journal.ppat.1009578)

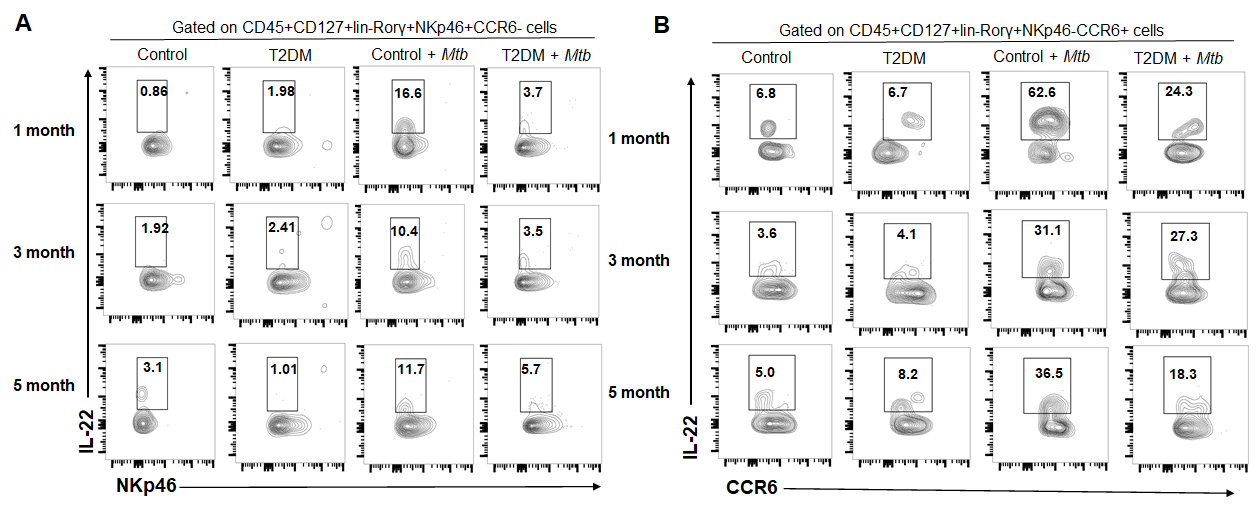

Supplement: S7 Fig — Control C57BL/6 and T2DM mice were infected with Mtb as shown in Fig 1 and described in the methods section. One, three and five months post Mtb infection lung single cell suspension was prepared and flowcytometry was performed. A representative flow cytometry figure for IL-22 producing (A) LTi and (B) NCR+ ILC3s is shown. (TIFF) [file ppat.1009578.s001.tiff]
